# An analysis of the burden of drug use disorders from 1990 to 2023: differences between China, India, and the United States

**DOI:** 10.3389/fphar.2026.1797092

**Published:** 2026-04-02

**Authors:** Meiling Hu, Hui Chen, Di Hu, Yan Zhang, Jincai Guo

**Affiliations:** 1 Department of Pharmacy, Changsha Stomatological Hospital, Changsha, China; 2 School of Stomatology, Hunan University of Chinese Medicine, Changsha, China; 3 School of Pharmacy, Hunan University of Chinese Medicine, Changsha, China

**Keywords:** deaths, disability-adjusted life years, drug use disorders, epidemiology, incidence, prevalence, trends

## Abstract

**Background:**

Drug use disorders (DUDs) represent a significant global health burden with marked regional variations. This study compares DUDs burden, trends, and projections among China, India, and the United States (US)—three countries comprising approximately 40% of the world’s population and spanning a socio-demographic index (SDI) range from 0.61 to 0.88—to elucidate how developmental stages influence DUDs patterns and inform targeted interventions.

**Methods:**

Data on the incidence, prevalence, deaths, and disability-adjusted life years (DALYs) of DUDs from 1990 to 2023 were extracted from the Global Burden of Disease Study 2023, stratified by sex, age group, and drug subtype. Temporal trends were analyzed via Joinpoint regression, and future burden to 2040 was projected using the Nordpred age-period-cohort model.

**Results:**

In 2023, the US had the highest age-standardized incidence rate (ASIR), age-standardized prevalence rate (ASPR), age-standardized mortality rate (ASMR), and age-standardized DALYs rate (ASDR), while India had the lowest. From 1990 to 2023, China showed declining trends across all age-standardized rate (ASR), in contrast to rising trends in the US; India showed mixed trends, with rises in ASIR, ASPR, and ASDR but a slight decrease in ASMR. The burden was concentrated in younger adults and men. Opioid use disorders contributed most to deaths and DALYs in all three countries. Projections from 2024 to 2040 indicate marked divergence: the US is expected to see growth across all indicators, with deaths nearly doubling (109.59%) and ASMR increasing by 90.76%; India is projected to experience rises in ASMR (30.40%) and ASDR (15.73%); while China shows modest increases across all ASR (4.61%–24.23%) despite a decline in incident cases (−7.45%).

**Conclusion:**

DUDs burden and trends differ markedly among the three countries. Emerging trends underscore the need for sustained, targeted interventions focusing on high-risk populations and specific drug types to mitigate future impacts.

## 1 Introduction

Drug use disorders (DUDs), as defined by the *Diagnostic and Statistical Manual of Mental Disorders* (DSM-5) and the *International Classification of Diseases* (ICD-11), are patterns of drug use—both illicit substances and non-medical use of prescription drugs—that cause significant impairment or harm (Grant and Chamberlain, 2016). These disorders present a major global public health issue, severely affecting physical and mental health, increasing suicide risk, psychiatric comorbidity, infectious diseases, and premature mortality (Degenhardt and Hall, 2012; Levitt et al., 2020; Zhang et al., 2024), while also imposing high socioeconomic costs through lost productivity, crime, and poverty (Degenhardt and Hall, 2012; Grant et al., 2016). Key risk factors for DUDs include young age, low education, and occupational status, emphasizing the social gradient of vulnerability (Swendsen et al., 2009). DUDs frequently co-occur with other substance use disorders (SUDs) and are strongly linked to mood, anxiety, and personality disorders (Compton et al., 2005; McCabe et al., 2008), highlighting complex psychiatric comorbidities. The growing non-medical use of prescription opioids and sedatives became a major public health issue in the United States (US) during the 1990s and early 2000s (Compton et al., 2005; McCabe et al., 2008), illustrating how pharmaceutical availability and prescribing practices can reshape drug epidemics. In 2021, over 53 million people globally were affected by DUDs, as reported by the Global Burden of Disease (GBD) study (Dongying et al., 2025), highlighting the need for continued research and monitoring. Decomposition analysis indicates that population growth was the primary contributor to the rise in DUDs-related disability-adjusted life years (DALYs) between 1990 and 2021, accounting for 35.31% of the increase. Additionally, the relationship between social development and the burden is complex. Countries with high socio-demographic index (SDI) scores, such as the US, exhibit abnormally high burdens, challenging the assumption of purely linear development (Dongying et al., 2025). Health inequality measurements confirm that the DUDs burden is rising globally and is increasingly concentrated in high-SDI countries. The US, in particular, stands out as a high-burden “outlier,” while China and India follow distinct patterns within their respective SDI groups (Dongying et al., 2025). Despite comprehensive global, regional, and national estimates from successive GBD studies (Amirkafi et al., 2024; Zhang et al., 2024; Dongying et al., 2025), few have systematically compared countries across the SDI spectrum or examined how social development influences long-term DUDs trends. Such comparisons are crucial for understanding diverse drivers of the DUDs burden and developing targeted prevention strategies.

This raises an important question: How do differing social development stages influence DUDs prevalence patterns within large populations? India, China, and the US, representing approximately 40% of the global population, span the SDI spectrum from 0.61 (India) to 0.73 (China) to 0.88 (The United States) in 2023 (GBD 2023, GBD 2025), making them an ideal comparative triangle. This design enables the exploration of how population size and social development interact to shape DUDs across different socioeconomic contexts. Moreover, epidemiological evidence indicates that drug subtypes—including opioids, amphetamines, cannabis, and cocaine—exhibit distinct epidemiological trajectories and health consequences (Compton et al., 2005; McCabe et al., 2008), suggesting that aggregated analyses may obscure important heterogeneity. Thus, this study uses the latest GBD 2023 data to compare long-term trends (1990–2023), projections to 2040, and age-sex distributions for DUDs incidence, prevalence, mortality, and DALYs in China, India, and the US from 1990 to 2023, with a particular focus on subtype-specific patterns. The goal is to reveal the heterogeneity of DUDs prevalence across these nations, providing a strong evidence base for developing targeted prevention and control strategies at both national and global levels.

## 2 Methods

### 2.1 Data sources

The GBD 2023 provides estimates of the burden caused by 375 diseases and injuries, as well as risk-attributable burden associated with 88 risk factors, including DUDs (GBD 2023 Disease and Injury and Risk Factor Collaborators, 2025). Data on the incidence, prevalence, deaths, and DALYs of DUDs from 1990 to 2023 for China, India, and the US, along with their corresponding 95% uncertainty intervals (UI), were obtained from the Global Health Data Exchange GBD Results Tool (https://vizhub.healthdata.org/gbd-results/). These data were stratified by sex, age, and drug category. The reporting of this study follows the Guidelines for Accurate and Transparent Health Estimates Reporting (GATHER) (Stevens et al., 2016). Due to the use of publicly available data, ethical review and informed consent were waived for this research.

### 2.2 Definition of DUDs

In the GBD 2023, DUDs were defined in accordance with the diagnostic criteria of the DSM-IV-TR or the ICD-10 (GBD 2023 Causes of Death Collaborators, 2025; GBD 2023 Disease and Injury and Risk Factor Collaborators, 2025). This category included amphetamine, cannabis, cocaine, and opioid use disorders, as well as other drug use disorders such as hallucinogen dependence, inhalant or solvent dependence, sedative dependence, and other forms of drug and substance dependence.

### 2.3 Statistical analysis

The burden of DUDs was evaluated by age group, sex, and drug category across China, India, and the US, with metrics including the number and age-standardized rate (ASR) of incidence, prevalence, deaths, and DALYs. All GBD estimates are presented as the mean of 250 distribution draws, with 95% UI defined by the 2.5th and 97.5th percentiles of these draws (GBD 2023 Disease and Injury and Risk Factor Collaborators, 2025). The UI reflects the estimates of uncertainty from model specification, stochastic variation, and measurement bias (Dongying et al., 2025). All rates were reported per 100,000 population, and ASR was calculated using the GBD 2023 world standard population. To examine temporal trends in the age-standardized incidence rate (ASIR), age-standardized prevalence rate (ASPR), age-standardized mortality rate (ASMR), and age-standardized DALYs rate (ASDR) of DUDs from 1990 to 2023, the average annual percent change (AAPC) was calculated via Joinpoint regression analysis (software version 5.2.0). A trend was defined as increasing if both the AAPC and its 95% confidence interval (CI) were greater than zero, or decreasing if both were less than zero; otherwise, the trend was considered stable.

Meanwhile, future trends in the burden of DUDs in China, India, and the US were projected using the Nordpred age-period-cohort model, which has been demonstrated to perform well in predicting future epidemiological trends (Arnold et al., 2020; Luo et al., 2023). Projections of the number and ASR for incidence, prevalence, deaths, and DALYs from 2024 to 2040 were generated, based on population projection data from the World Population Prospects 2024 revision (https://population.un.org/wpp/). To assess the robustness of the projected estimates against potential data variations, a sensitivity analysis was conducted using the lower and upper bounds of the dataset.

All statistical analyses and visualizations were conducted in R software (version 4.4.1), employing the “Nordpred” and “ggplot2” packages for predictive modeling and data visualization, respectively. A p-value <0.05 was considered statistically significant.

## 3 Results

### 3.1 Descriptive analysis of the burden of DUDs

**FIGURE 1:**
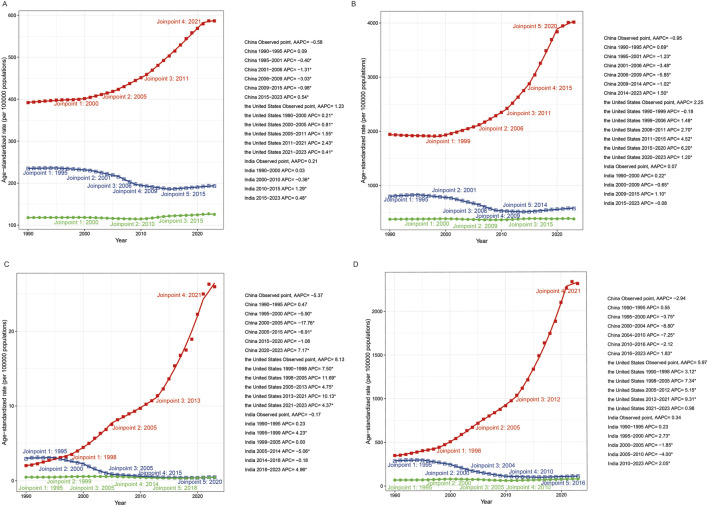
The joinpoint regression analysis of the age-standardized rate (ASR) of incidence **(A)**, prevalence **(B)**, deaths **(C)** and disability-adjusted life years (DALYs) **(D)** of drug use disorders in China, India, and the United States.

In 2023, marked disparities in the burden of DUDs were observed across the three countries (Table 1). China reported the highest number of incident cases, while the US had the highest ASIR as well as the highest counts and ASR of prevalence, deaths, and DALYs, reflecting a more severe DUDs burden. India recorded the lowest ASIR and the smallest numbers and ASR of prevalence, deaths, and DALYs, a pattern that may be partially attributable to underreporting. Trends from 1990 to 2023 reveal sharply divergent trajectories. China achieved substantial reductions across all metrics, with deaths and DALYs declining by more than 50%. In sharp contrast, the US experienced dramatic increases, with a 15-fold rise in deaths and a sevenfold rise in DALYs, and all ASR climbing persistently, underscoring a deepening and increasingly lethal national epidemic. India presented a mixed pattern: incident cases, prevalent cases, deaths, and DALYs approximately doubled, and ASIR, ASPR, and ASDR increased, while ASMR showed a slight decline, indicating a growing but still moderately severe DUDs challenge. Detailed temporal trend trajectories and specific turning points identified via Joinpoint regression analysis are presented in Figure 1.

**TABLE 1:** Number of cases and ASR of DUDs incidence, prevalence, deaths and DALYs in 1990 and 2023, with AAPC from 1990 to 2023.

Measure	Location	1990		2023		AAPC (95%CI) 1990–2023	P
		Number (95%UI)	ASR (95%UI)	Number (95%UI)	ASR (95%UI)		
Incidence	China	3132505 (2677301–3718527)	234.91 (201.45–277.6)	2720246 (2283703–3307063)	193.24 (163.16–232.63)	−0.58 (−0.6 to −0.57)	<0.001
	India	959896 (793857–1179201)	117.96 (98.62–143.78)	1981112 (1657011–2391424)	125.57 (105.53–150.72)	0.21 (0.19–0.23)	<0.001
	The United States	1002844 (825863–1196157)	391.88 (324.47–472.75)	1766916 (1546760–2019922)	586.5 (509.68–676.11)	1.23 (1.21–1.24)	<0.001
Prevalence	China	11134940 (9429335–13498769)	797.3 (684.42–955.92)	7348874 (6193406–8625108)	576 (480.78–695.53)	−0.95 (−0.99 to −0.91)	<0.001
	India	3109216 (2507499–3875550)	376.68 (306.56–461.33)	6073948 (4923324–7188637)	382.42 (311.04–451.57)	0.07 (0.04–0.1)	<0.001
	The United States	5110069 (4312195–5970003)	1944.09 (1631.96–2314.01)	12796839 (11594263–14140490)	4016.38 (3594.04–4478.6)	2.25 (2.2–2.29)	<0.001
Deaths	China	34858 (24349–47785)	2.93 (2.06–4.02)	8186 (5560–11713)	0.47 (0.32–0.68)	−5.37 (−5.54 to −5.2)	<0.001
	India	3042 (2010–4334)	0.47 (0.31–0.67)	6238 (4376–9174)	0.44 (0.31–0.64)	−0.17 (−0.31 to −0.03)	0.02
	The United States	5785 (4351–7411)	2.02 (1.52–2.59)	95333 (76054–115275)	25.97 (20.79–31.24)	8.13 (7.94–8.35)	<0.001
DALYs	China	3757896 (2856700–4705940)	284.48 (218.4–355.38)	1502237 (1122849–1870840)	107.6 (78.68–134.49)	−2.94 (−3.02 to −2.86)	<0.001
	India	528747 (388114–679620)	67.86 (49.67–86.95)	1184024 (904700–1449828)	75.8 (58.26–92.5)	0.34 (0.28–0.4)	<0.001
	The United States	963774 (744832–1144023)	349.08 (269.92–415.96)	7796439 (6528569–9173050)	2312.52 (1933.51–2717.54)	5.97 (5.89–6.05)	<0.001

AAPC, average annual percentage change; ASR, age-standardized rate; DUDs, drug use disorders; DALYs, disability-adjusted life years.

### 3.2 Age and sex disparities in the burden of DUDs

**FIGURE 2:**
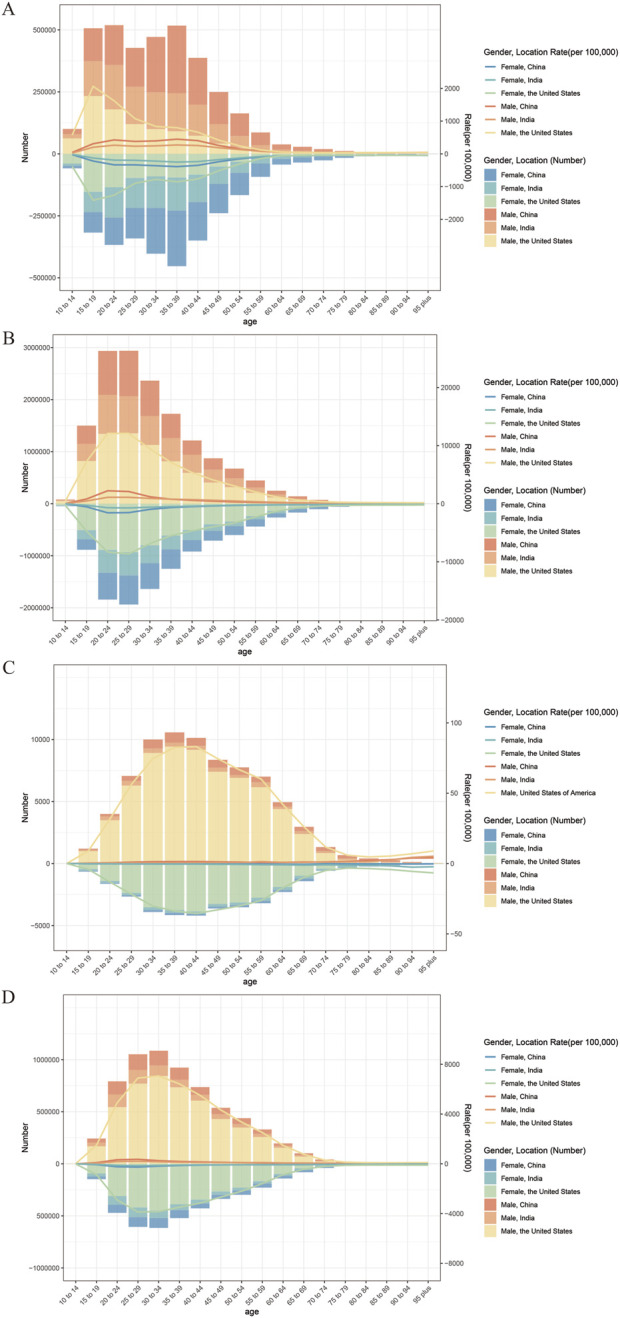
Case numbers and rate for the incidence **(A)**, prevalence **(B)**, deaths **(C)**, and disability-adjusted life years (DALYs) **(D)** of drug use disorders, by age group and sex, in 2023.

The age distribution of the burden of DUDs was similar across China, India, and the US, with the burden primarily concentrated in younger adults (Figure 2). In 2023, the highest number and ASR of incidence, prevalence, and DALYs occurred in the 20–39 years in China and India, but shifted to the 15–34 years in the US (Supplementary Tables S1-S3). The highest number of deaths was observed in the 35–39 age group in China and the US, compared to 55–59 in India. Both China and India reported the highest ASMR in the 90+ age group, while the US peaked in the 40–44 age group. From 1990 to 2023, ASIR, ASPR, and ASDR generally declined across most age groups in China, and ASMR decreased in all age groups (Supplementary Figure S1). In contrast, trends in India were more varied: ASIR rose in most age groups, while ASPR, ASMR, and ASDR showed mixed patterns of increase and decrease. The US demonstrated the most consistent upward trend, with nearly all age groups experiencing increases in all four ASR metrics.

When stratified by sex, the burden of DUDs was consistently higher in men than in women across all three countries (Figure 2; Supplementary Table S4). However, sex-specific trends differed markedly. In China, all ASR declined for both sexes, with a greater reduction observed in women. In contrast, all ASR increased for both sexes in the US, and the relative increase was larger among women. India presented a more heterogeneous pattern: ASIR and ASDR rose in both sexes; ASPR increased in women but decreased in men; and ASMR declined in both sexes (Supplementary Table S4).

### 3.3 Burden and temporal trends by type of DUDs

The burden distribution across drug subtypes revealed both shared patterns and distinct profiles among the three countries. In common, all three countries had the highest number and ASR of incidence for other DUDs, and the lowest for cocaine use disorders. Additionally, opioid use disorders (OUDs) showed the highest number and ASR of deaths and DALYs in each country (Supplementary Table S5). In terms of differences, China had the highest number and ASR of prevalence for amphetamine use disorders. India reported the greatest number and ASR of prevalence for cannabis use disorders, followed by OUDs. In contrast, the US had the highest number and ASR of prevalence, deaths, and DALYs for OUDs.

Trends from 1990 to 2023 exhibit more pronounced divergence. In China, ASIR, ASPR, ASMR, and ASDR showed declining trends for most subtypes, except for cannabis use disorders (Supplementary Table S5). India showed a mixed trend: cannabis and cocaine use disorders had gradual declines in ASIR and ASPR, while the other three subtypes increased slowly. Notably, ASDR for amphetamine and opioid use disorders rose. Conversely, the US had increases in ASIR, ASPR, and ASDR for nearly all subtypes, except for a slight decline in cannabis use disorders. The ASPR for OUDs rose markedly, and ASMR increased across all subtypes, with the sharpest rise for amphetamine use disorders.

### 3.4 Predictive analysis

**FIGURE 3:**
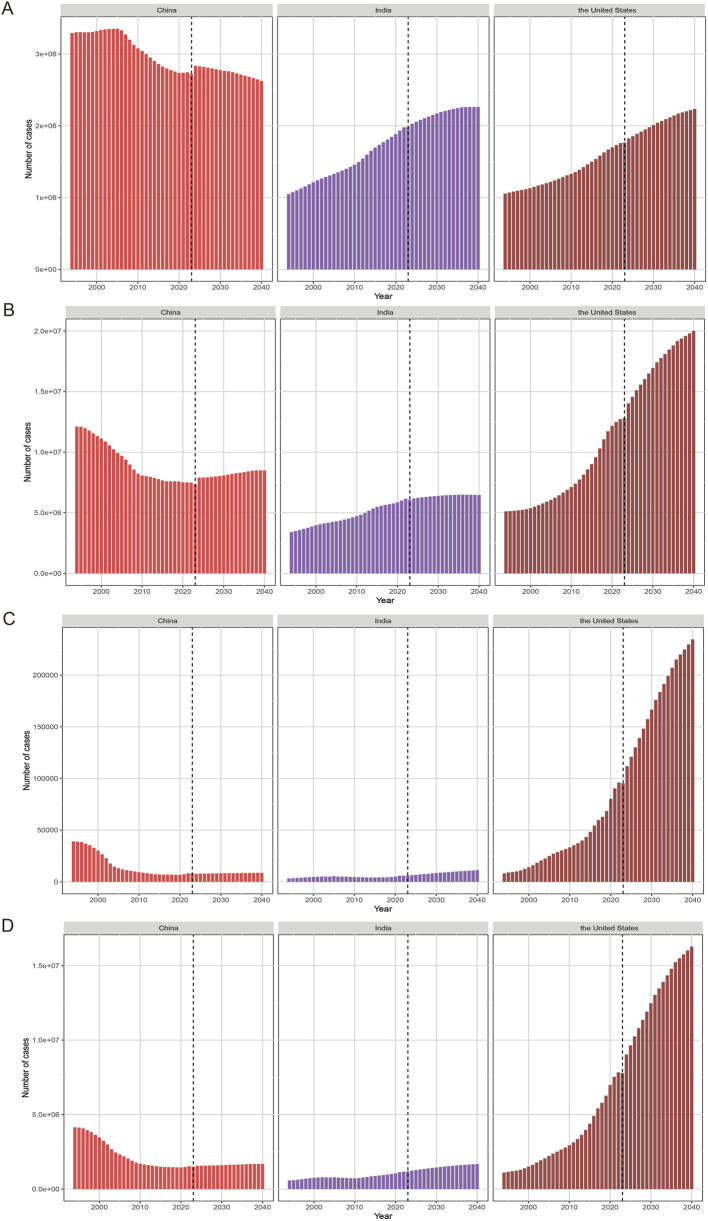
The projected case numbers of incidence **(A)**, prevalence **(B)**, deaths **(C)** and disability-adjusted life years (DALYs) **(D)** for drug use disorders through 2040.

**FIGURE 4:**
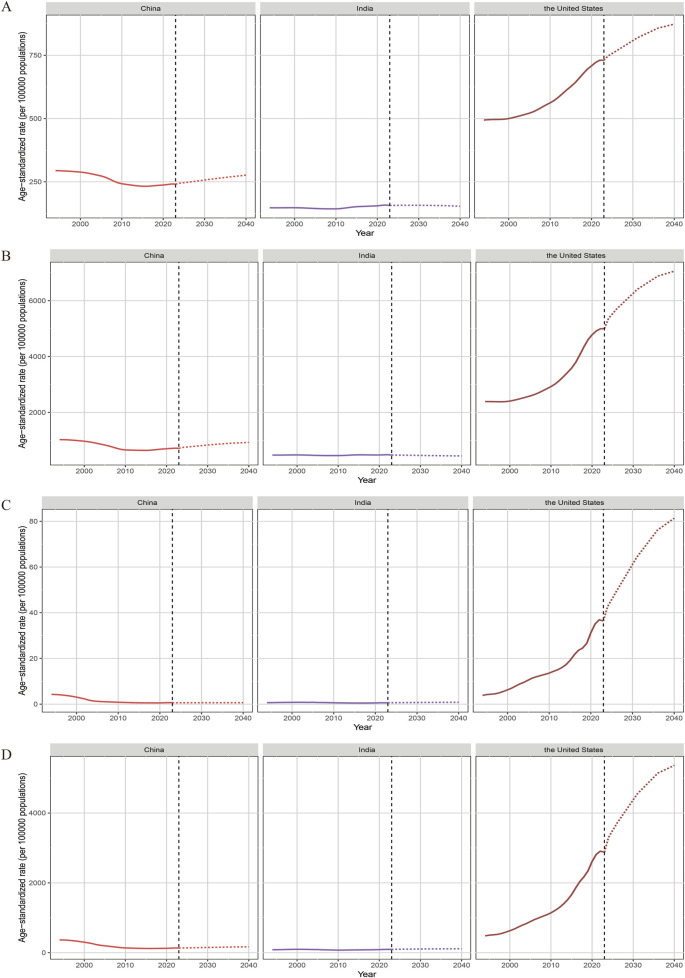
The projected age-standardized rate (ASR) of incidence **(A)**, prevalence **(B)**, deaths **(C)** and disability-adjusted life years (DALYs) **(D)** for drug use disorders through 2040.

Projections from 2024 to 2040 indicated divergent trends across the three countries (Figures 3, 4). In China, the number of incident cases was projected to decline by 7.45%, while ASIR was expected to increase by 13.04%. Both the number and ASR for prevalence, deaths, and DALYs were anticipated to rise, with ASDR showing the most pronounced increase (24.23%), followed by ASPR (23.72%) (Supplementary Tables S6-S9). For India, the numbers of incident cases, prevalent cases, deaths, and DALYs were projected to increase by 11.57%, 4.63%, 73.14%, and 35.57%, respectively. However, its ASR presented a mixed pattern: ASIR and ASPR showed slight declines (−2.10% and −6.67%, respectively), while ASMR and ASDR were predicted to increase by 30.40% and 15.73%. The US was projected to experience the most dramatic growth across all indicators. Notably, the number and ASR for deaths were expected to nearly double (109.59% and 90.76%, respectively), followed by substantial increases in ASDR (62.48%) and ASPR (31.48%). Sensitivity analyses using data bounds showed similar predicted trends, supporting the reliability of the projections (Supplementary Tables S10-S11).

## 4 Discussion

This study highlights a significant and systematic epidemiological divergence in DUDs among China, India, and the US from 1990 to 2023. These differences are evident in the dominant drugs: the US faces a high-fatality opioid crisis, China has shifted to a high-disability issue dominated by amphetamine-type stimulants, and India grapples with a dual burden of widespread cannabis use and regional opioid problems. Demographically, the burden in all three countries primarily affects the working-age population. However, the US exhibits an age pattern marked by “earlier onset” (ages 15–34) and “earlier mortality” (ages 35–44). Gender differences also vary: the US demonstrates “accelerated progression” among women, while in China, the burden decreases for both men and women, with a more significant reduction in women. The trend in India is more complex due to data gaps and social factors. Looking ahead to 2040, the US is projected to experience worsening outcomes across all indicators, India faces a risk of sharply rising mortality, and China anticipates a decline in the number of incidence but continued increases in other metrics. These systemic differences suggest that DUDs prevalence is influenced by each nation’s unique “material-social-policy” ecosystem. Understanding this diversity is crucial for moving beyond generalized strategies and implementing targeted interventions.

The DUDs burden in the US presents as a significant and evolving public health crisis, with OUDs being the primary driver of the country’s high mortality and disability rates. All ASR have remained at alarmingly high levels through 2023. This crisis is often characterized as “illegal synthetic opioid-driven,” a description aligned with the Lancet Commission on Regional Health in the Americas’ diagnosis of “multisystem regulatory failure,” where drugs like fentanyl are far more lethal than traditional opioids (The Lancet Regional, 2023). A key finding of this study is that the COVID-19 pandemic acted as an unexpected “natural experiment.” Joinpoint analysis reveals that multiple ASR for DUDs in the US reached growth inflection points in 2020–2021, with growth rates significantly slowing. This shift likely resulted from the temporary convergence of two factors: social distancing measures disrupting substance distribution networks and the public health emergency prompting greater awareness of risks and harm reduction efforts (Miech et al., 2021). However, these trends should be seen as temporary fluctuations within a broader upward trajectory. The SOURCE (Simulation of Opioid Use, Response, Consequences, and Effects) system dynamics model suggests that the opioid crisis in the US is driven by a complex interaction among social influence, risk perception, treatment capacity, and changes in the supply of both prescription and illicit opioids. Model quantification shows that illicitly synthesized opioids, especially fentanyl, are the dominant drivers of mortality, far surpassing interventions like naloxone (Lim et al., 2022). This further supports the conclusion that the observed inflection points are temporary shocks, not reversals, in a persistent upward trend. In the US, only 13.0% of individuals with past-year SUDs, including alcohol and drugs, received any treatment between 2016 and 2019 despite substantial need (Sahker et al., 2024). This persistent treatment gap highlights the limits of even well-resourced health systems. Comparable national estimates are unavailable for China and India. However, evidence from low- and middle-income countries, including India, indicates even lower treatment coverage, with major barriers such as low perceived need, limited awareness of services, financial constraints, and stigma (Sarkar et al., 2021). Global research output on SUDs—commonly used as a proxy for DUDs-focused research—is heavily concentrated in the US, which accounts for the majority of publications in this field. China is the only Asian country among the top 10 by first-author output, while India’s contribution remains modest (Tran et al., 2019). This imbalance may reflect differences in treatment infrastructure and research capacity. To address this crisis, the US must focus on disrupting the illegal fentanyl supply chain while scaling up harm reduction measures to match the crisis’s severity.

In contrast, China has successfully reduced its DUDs burden, demonstrating a “strategic transformation under effective control.” Between 1990 and 2023, all age-standardized burden indicators in China showed a significant decline, validating the success of past anti-drug policies. Key control measures include the establishment of the National Narcotics Control Commission (NNCC) in 1990, which coordinates enforcement across more than 40 departments. The State Council issued the *Regulations on the Control of Narcotic Drugs and Psychotropic Substances* in 2005 and the *Regulations on the Control of Non-medical Narcotic Drugs and Psychotropic Substances* in 2015. The list of controlled substances expanded from 31 in 1979 to 509 in 2024, including the blanket scheduling of all fentanyl-related and synthetic cannabinoid–related substances (Wang et al., 2025). However, drug use patterns have shifted, with amphetamine use disorder now being the most prevalent. Methamphetamine use surpassed traditional opioids in 2014 and has continued to rise. By 2023, China had 896,000 active drug users, with 455,000 abusing methamphetamine (Office of the National Narcotics Control Commission, 2024). This shift reflects a transition from a “high-fatality crisis” driven by opioids to a “high-disability” challenge caused by synthetic drugs. Wastewater epidemiology studies highlight spatial heterogeneity and poly-drug abuse patterns across China (Liu et al., 2021), while research from Hong Kong shows increased methamphetamine-related clinical severity during the pandemic (Lam, 2023). These changes point to evolving drug use patterns that are increasing health risks and straining healthcare systems. Consequently, China’s prevention and control strategy must evolve from “high-pressure drug prohibition” to a more nuanced approach focused on “precision prevention and control” and recovery support, emphasizing synthetic drugs, chronic addiction management, and building capacity for complex clinical cases. India is at a critical juncture in its drug use epidemic. Although its DUDs burden remains relatively low, it is rising, with cannabis use disorder being the most prevalent. This reflects the widespread use of cannabis in India (Ministry of Social Justice and Empowerment, Government of India, 2019). However, a more severe challenge lurks beneath the surface: approximately 2.1% of India’s population used opioids in 2019, with heroin being the primary driver of this crisis (Singh and Rao, 2021). In regions like Punjab, large populations of inadequately treated heroin-dependent individuals exhibit rampant injecting drug use and high-risk behaviors, yet treatment access remains extremely limited (Avasthi et al., 2019). Moreover, with changes in narcotics control regulations and the international pharmaceutical market, India faces the looming risk of a prescription opioid epidemic (Singh and Rao, 2021). India’s control framework is anchored in the *Narcotic Drugs and Psychotropic Substances Act, 1985* (NDPS Act), which criminalizes trafficking and consumption and imposes tiered penalties. The Narcotics Control Bureau (NCB) coordinates seizures and maintains the NIDAAN database of offenders. The *National Action Plan for Drug Demand Reduction* (NAPDDR, 2019) funds 46 Addiction Treatment Facilities (ATFs) and Integrated Rehabilitation Centres for Addicts (IRCAs). Harm reduction measures include needle and syringe exchange and opioid substitution therapy delivered through the National AIDS Control Organization (Singh and Chadda, 2025). In summary, India stands at a critical “window of opportunity” for intervention, requiring proactive policy responses to guard against the potential prescription opioid wave and urgently expand evidence-based treatment services.

The epidemiological burden of DUDs significantly impacts socioeconomic structures, with peak incidence, prevalence, and disability burden concentrated in the core working-age population (ages 20–39) in China, India, and the US. This commonality highlights that DUDs are not merely individual health issues but major public health challenges that undermine national human capital and economic productivity. However, the three countries display distinct age-related risk patterns for DUDs. In the US, the crisis manifests as a dual compression of “early onset” and “early mortality.” Our study shows that the peak incidence and prevalence of DUDs in the US occur in the 15–34 age group, likely driven by heightened addiction susceptibility in adolescents. National data suggest that individuals who try an illegal drug before age 15 are 6.5 times more likely to develop DUDs compared to those who start at age 21 or later (National Center for Drug Abuse Statistics, 2025). Additionally, adolescents (ages 12–17) who initiate cannabis or prescription drug abuse exhibit a higher prevalence of DUDs within 12 months than young adults (ages 18–25), indicating that early exposure accelerates addiction progression (Volkow et al., 2021). This rapid escalation leads to an early and substantial disease burden. Closely tied to early onset is early mortality. In the US, the peak mortality rate for DUDs occurs during the prime working years of 35–44, driven by the lethal effects of synthetic opioids like fentanyl. However, despite the youth-centered nature of this crisis, the treatment system suffers from a significant “response delay” (Pilarinos et al., 2022). For instance, nearly 70% of young individuals (ages 13–22) who experienced a nonfatal opioid overdose received no addiction treatment in the month following the overdose, and only 2% received medication-assisted treatment (MOUD) (Alinsky et al., 2020). This systemic delay in treatment contributes to widespread premature mortality among the most productive age groups. In contrast, data from China and India show a different pattern, with ASMR peaks occurring in the 90+ age cohort. While this finding may initially suggest elevated mortality risk among the elderly, it should be interpreted cautiously due to a likely denominator effect stemming from small population sizes in the oldest age groups. Nonetheless, even a limited number of deaths in this vulnerable population highlights the importance of comprehensive lifecycle health management and continued monitoring of DUDs-related harms across all ages. In summary, the age distribution of DUDs raises an alarm for all three nations, but response strategies must be tailored to each country’s specific age-related burden. Effective interventions require advancing timelines for treatment and ensuring that therapeutic responses are aligned with the spatiotemporal distribution of at-risk populations.

The gender dimension of DUDs shows significant variation across China, India, and the US, influenced by both biological and socio-ecological factors. Women may be more vulnerable to certain substances due to the “telescope effect,” where addiction progresses more rapidly (Kliewer et al., 2022). This study identified three distinct gender trajectories, highlighting the role of socio-ecological and policy environments in shaping gender-specific patterns. In the US, the DUDs burden rises for both genders, but the female burden progresses at a faster rate. One factor likely playing a significant role is that women are more likely to have chronic pain, for which they are prescribed opioids at higher doses and longer-term use compared with men. Furthermore, women are more likely to “doctor shop” to obtain controlled substances, an issue that may be intertwined with the higher prevalence of chronic pain (Ait-Daoud et al., 2019). According to the 2024 National Survey on Drug Use and Health (NSDUH), women accounted for 46.4% of illicit drug users aged 12 and older in the past year, 42.9% of individuals with DUDs, and 45.0% of those receiving treatment for drugs (Substance Abuse and Mental Health Services Administration, 2025). These data indicate that the gender gap in treatment accessibility is narrowing. However, barriers remain, such as fear of punitive consequences like criminal justice involvement, child welfare intervention, and loss of custody rights (National Institute on Drug Abuse, 2020), as well as the treatment system’s failure to integrate childcare and pregnancy care (Meyer et al., 2019). These factors contribute to the accelerated progression of DUDs among women in the US. In contrast, China has seen significant declines in DUDs burden for both genders, with women experiencing a more pronounced reduction. However, the shift from traditional opioids to synthetic drugs like methamphetamine is altering gender-specific risk patterns (Zhang and Demant, 2021). Interestingly, women’s vulnerability to relapse in China is linked to emotional trust within families, and clinical evidence suggests that mood disorders and self-medication for emotional distress are key drivers for female drug use (Zhang et al., 2013). China’s focus should now shift to addressing these psychosocial vulnerabilities in the context of synthetic drug use. India presents a more complex situation due to significant data gaps. Rising drug use burdens for both genders are often hidden by historical underreporting of female drug use, which has been linked to structural issues like gender inequality and high-risk occupations such as sex work (Lal et al., 2015). These factors contribute to the marginalization of vulnerable women and hinder effective public health responses. India’s challenge lies in making these hidden risks visible and ensuring women’s needs are addressed in national drug policies. These divergent trajectories collectively underscore the imperative to transcend male-centered traditional prevention paradigms and shift toward more gender-sensitive public health response strategies.

The substantial divergences in the burden trends of DUDs subtypes across China, India, and the US can be partially attributed to the distinct pharmacological properties of each drug category, which fundamentally determine their addictive potential, toxicological features, and subsequent health outcomes. This is particularly evident for OUDs. As potent μ-opioid receptor agonists, synthetic opioids such as fentanyl exhibit ultra-high efficacy and affinity, producing intense euphoria at minute doses while profoundly suppressing brainstem respiratory centers—a lethal combination compounded by an extremely narrow therapeutic window (Comer and Cahill, 2019). Chronic use triggers receptor desensitization and downregulation, leading to tolerance (requiring dose escalation) and dependence (withdrawal-driven negative reinforcement) (Mathis et al., 2025). These characteristics underpin the substantial contribution of OUDs to deaths and DALYs across all three countries. This pattern is most pronounced in the US, where a deeply entrenched illicit fentanyl supply has driven a disproportionate rise in all key indicators of OUDs (Ciccarone, 2019). The potent sympathomimetic effects of amphetamine-type stimulants (ATS), primarily due to excessive catecholamine release, are known to cause severe cardiovascular toxicity (e.g., hypertension, arrhythmias, myocardial injury) and lasting neuropsychiatric damage (Darke et al., 2008; Cruickshank and Dyer, 2009; Neumann et al., 2024). This pharmacological mechanism provides a strong biological basis for the significant health burdens associated with amphetamine use at the population level. Consistent with this, our results show rising mortality and disability from amphetamine use disorders in India, alongside a sharp increase in the ASMR in the US. This trend is supported by Centers for Disease Control and Prevention (CDC) data, which report a continued sharp rise in overdose deaths involving psychostimulants, including methamphetamine, in 2022 (Centers for Disease Control and Prevention, 2023). Furthermore, the co-use of methamphetamine with opioids creates a synergistic risk profile: cardiovascular stress compounds respiratory depression, complicating clinical management and further elevating mortality (Ciccarone, 2021; Daniulaityte et al., 2020; The, 2018). Cannabis use disorders, by contrast, involve mild modulation of the endocannabinoid system, characterized by slow dependence development, minimal acute physiological toxicity, and low direct mortality (Hall and Degenhardt, 2014; Weinstein and Gorelick, 2011; Hoch et al., 2025). This aligns with the declining trends in cannabis use disorders-related metrics observed in India and the US. These pharmacological insights underscore the imperative for drug-specific public health strategies. Tailoring interventions to the pharmacological realities of each drug type—such as expanding naloxone access for opioids and implementing cardiovascular monitoring for stimulant users—is essential to mitigate the evolving burden of DUDs.

Projections for 2040 highlight starkly different challenges for the US, India, and China. In the US, all DUDs burden metrics are expected to worsen, signaling a deepening crisis driven by illicit synthetic opioids. The SOURCE model indicates that future mortality rates are highly sensitive to “further penetration of illicit synthetic opioids into the supply chain,” with the risk of contamination spreading to other drug markets potentially “driving sustained mortality growth” (Lim et al., 2022). Thus, dismantling the fentanyl supply chain must be prioritized, alongside expanding harm reduction and treatment networks. Without these measures, regulation and treatment alone cannot reverse the trend. In India, a projected 73.14% increase in deaths vastly outpaces the growth in incident cases and DALYs, strongly indicating a future surge in lethality. This is grounded in India’s existing heroin dependence, widespread injection drug use, and significant treatment gaps (Avasthi et al., 2019), compounded by revisions to narcotics regulations that increase the risk of prescription opioid abuse (Singh and Rao, 2021). These factors make India highly vulnerable to a rise in deaths, either from the influx of potent synthetic opioids like fentanyl or the health collapse of untreated individuals. India’s immediate priority should be to establish a real-time overdose death monitoring system, using it to guide emergency interventions and addiction treatment capacity building in primary healthcare. China’s outlook presents a structural contradiction: while new cases have slightly decreased, other metrics continue to rise. This reflects the epidemiological shift from heroin to synthetic drugs like methamphetamine, which cause more severe long-term neuropsychiatric damage and comorbidities (Darke et al., 2008). Consequently, China’s strategy must evolve from focusing on reducing new cases to addressing the growing disease burden. This requires investment in community-based rehabilitation and long-term support systems to manage the chronic impact of synthetic drug addiction.

This study has several limitations. First, the GBD 2023 estimates rely on older diagnostic criteria (DSM-IV-TR/ICD-10), while current clinical practice uses DSM-5/ICD-11, which may affect diagnostic comparability (First et al., 2022). Second, data quality varies across countries, with underreporting in regions like India and China—especially for non-fatal outcomes—likely leading to an underestimation of DUDs burden in these countries compared to the US. Finally, projections are based on pre-2023 data and may not fully capture rapidly evolving trends, such as the spread of synthetic opioids or new psychoactive substances. Consequently, these forecasts should be viewed as trend-based illustrations, highlighting the need for real-time surveillance to inform policy.

## 5 Conclusion

This study demonstrates that the global burden of DUDs is shaped by each nation’s unique “substance-society-policy” triad. The US’s experience highlights the devastating impact of illicit synthetic opioids when regulation fails. China’s situation underscores the effectiveness of strict controls, while also revealing the pressures of emerging drug challenges. Meanwhile, India’s context emphasizes the urgent need for proactive interventions before an epidemic escalates. Projections through 2040 reflect current trajectories, not predetermined outcomes, and serve as a reminder of the need for precision governance. Policymakers must adopt a complex systems approach that respects national differences while addressing core drivers to guide countries toward meaningful health improvements.

## Data Availability

The datasets used in this study are all publicly available (https://vizhub.healthdata.org/gbd-results/), further inquiries can be directed to the corresponding author.
