# Supplementary material for: An analysis of the burden of drug use disorders from 1990 to 2023: differences between China, India, and the United States
Source: Front Pharmacol. 2026 Apr 2;17:1797092. doi: 10.3389/fphar.2026.1797092 (PMC13083133; doi:10.3389/fphar.2026.1797092)

**An analysis of the burden of drug use disorders from 1990 to 2023: Differences between China, India, and the United States**

**Meiling Hu, Hui Chen, Di Hu, Yan Zhang, Jincai Guo**

Table of contents

[Supplementary Table S1 1](#_Toc223519599)

[Supplementary Table S2 6](#_Toc223519600)

[Supplementary Table S3 11](#_Toc223519601)

[Supplementary Table S4 16](#_Toc223519602)

[Supplementary Table S5 18](#_Toc223519603)

[Supplementary Table S6 23](#_Toc223519604)

[Supplementary Table S7 26](#_Toc223519605)

[Supplementary Table S8 29](#_Toc223519606)

[Supplementary Table S9 32](#_Toc223519607)

[Supplementary Table S10. 35](#_Toc223519608)

[Supplementary Table S11 41](#_Toc223519609)

[Supplementary Figure S1 53](#_Toc223519610)

Supplementary Table S1**.** Number of cases and ASR of DUDs incidence, prevalence, deaths and DALYs in China (1990 vs. 2023), stratified by age, with AAPC from 1990 to 2023.

| **Measure** | **Age group** | **1990** | | **2023** | | **AAPC (95%CI)** **1990–2023** | **P** |
| --- | --- | --- | --- | --- | --- | --- | --- |
|  |  | **Number (95%UI)** | **ASR (95%UI)** | **Number (95%UI)** | **ASR (95%UI)** |  |  |
| **Incidence** | 10-14 | 24789  (4622-58658) | 24.17  (4.51-57.2) | 30998  (5612-73714) | 34.92  (6.32-83.04) | 1.13 (1.09 to 1.17) | <0.001 |
|  | 15-19 | 449213  (360712-566131) | 353.71  (284.02-445.77) | 215249  (163317-274665) | 267.19  (202.73-340.94) | -0.84 (-0.89 to -0.8) | <0.001 |
|  | 20-24 | 722182  (568063-890974) | 545.57  (429.15-673.09) | 271573  (208476-335680) | 383.81  (294.64-474.42) | -1.03 (-1.06 to -1) | <0.001 |
|  | 25-29 | 489145  (376943-628619) | 443.98  (342.14-570.57) | 280283  (203042-365105) | 359.38  (260.34-468.15) | -0.63 (-0.65 to -0.61) | <0.001 |
|  | 30-34 | 379183  (286414-496369) | 428.45  (323.63-560.87) | 408163  (307303-538925) | 379.98  (286.08-501.71) | -0.35 (-0.36 to -0.34) | <0.001 |
|  | 35-39 | 437570  (308797-601131) | 477.88  (337.25-656.51) | 496876  (348224-704467) | 420.3  (294.56-595.9) | -0.38 (-0.39 to -0.37) | <0.001 |
|  | 40-44 | 290321  (190936-396625) | 431.46  (283.76-589.44) | 343658  (220509-481059) | 377.87  (242.46-528.95) | -0.4 (-0.41 to -0.38) | <0.001 |
|  | 45-49 | 148196  (104600-201525) | 286.33  (202.1-389.37) | 247214  (175998-342199) | 247.85  (176.45-343.07) | -0.43 (-0.44 to -0.42) | <0.001 |
|  | 50-54 | 84679  (59493-117248) | 177.01  (124.36-245.09) | 181245  (119558-258014) | 153.08  (100.98-217.92) | -0.43 (-0.45 to -0.42) | <0.001 |
|  | 55-59 | 44135  (34973-57361) | 101.46  (80.4-131.87) | 104581  (81900-134338) | 88.46  (69.28-113.63) | -0.41 (-0.43 to -0.4) | <0.001 |
|  | 60-64 | 22588  (17420-28477) | 63.71  (49.14-80.33) | 43640  (33278-54116) | 53.59  (40.87-66.46) | -0.52 (-0.53 to -0.51) | <0.001 |
|  | 65-69 | 17429  (13910-21203) | 63.58  (50.75-77.35) | 36991  (29844-43970) | 48.47  (39.11-57.62) | -0.81 (-0.83 to -0.79) | <0.001 |
|  | 70-74 | 11752  (9426-14596) | 62.26  (49.93-77.32) | 27367  (21560-33811) | 44.82  (35.31-55.38) | -0.98 (-1 to -0.96) | <0.001 |
|  | 75-79 | 6785  (5088-8449) | 59.37  (44.53-73.94) | 15641  (11268-19691) | 42.52  (30.63-53.53) | -1 (-1.02 to -0.97) | <0.001 |
|  | 80-84 | 3201  (2346-4018) | 57.33  (42.02-71.95) | 9306  (6559-11752) | 41.42  (29.2-52.32) | -0.97 (-0.99 to -0.95) | <0.001 |
|  | 85-89 | 1102  (900-1354) | 55.19  (45.1-67.82) | 5119  (4079-6224) | 41.05  (32.7-49.91) | -0.89 (-0.91 to -0.87) | <0.001 |
|  | 90-94 | 201  (154-253) | 53.32  (41.01-67.1) | 1838  (1399-2272) | 40.6  (30.89-50.18) | -0.81 (-0.83 to -0.79) | <0.001 |
|  | 95+ | 34  (23-45) | 52.25  (35.18-69.26) | 503  (323-657) | 40.5  (26.03-52.92) | -0.77 (-0.78 to -0.75) | <0.001 |
| **prevalence** | 10-14 | 20235  (3076-49095) | 19.73  (3-47.87) | 25465  (3838-61676) | 28.69  (4.32-69.48) | 1.15 (1.11 to 1.19) | <0.001 |
|  | 15-19 | 1010444  (740657-1345527) | 795.62  (583.19-1059.46) | 549718  (356063-809029) | 682.36  (441.98-1004.25) | -0.45 (-0.49 to -0.41) | <0.001 |
|  | 20-24 | 3675057  (2837744-4859556) | 2776.34  (2143.79-3671.17) | 1358754  (1026924-1775831) | 1920.33  (1451.36-2509.79) | -1.12 (-1.18 to -1.07) | <0.001 |
|  | 25-29 | 2909040  (2375489-3630593) | 2640.41  (2156.13-3295.33) | 1430640  (1122886-1797678) | 1834.39  (1439.79-2305.02) | -1.07 (-1.11 to -1.03) | <0.001 |
|  | 30-34 | 1277693  (1031693-1628805) | 1443.72  (1165.75-1840.45) | 1170033  (920052-1517788) | 1089.23  (856.52-1412.97) | -0.83 (-0.86 to -0.8) | <0.001 |
|  | 35-39 | 842283  (691932-1048201) | 919.88  (755.68-1144.77) | 845142  (669058-1107284) | 714.9  (565.95-936.64) | -0.74 (-0.77 to -0.71) | <0.001 |
|  | 40-44 | 477165  (394478-584466) | 709.13  (586.25-868.6) | 493547  (390389-625612) | 542.68  (429.25-687.89) | -0.79 (-0.82 to -0.76) | <0.001 |
|  | 45-49 | 290255  (238855-358034) | 560.81  (461.49-691.76) | 406747  (321001-505035) | 407.79  (321.82-506.33) | -0.96 (-0.98 to -0.93) | <0.001 |
|  | 50-54 | 213022  (167809-259685) | 445.3  (350.78-542.84) | 359352  (278115-448186) | 303.51  (234.9-378.55) | -1.17 (-1.2 to -1.13) | <0.001 |
|  | 55-59 | 153936  (121181-181796) | 353.89  (278.59-417.94) | 276094  (211278-333882) | 233.54  (178.71-282.42) | -1.24 (-1.27 to -1.21) | <0.001 |
|  | 60-64 | 100869  (80139-123340) | 284.52  (226.05-347.91) | 150264  (114191-188343) | 184.54  (140.23-231.3) | -1.29 (-1.32 to -1.26) | <0.001 |
|  | 65-69 | 70970  (57325-85571) | 258.91  (209.13-312.17) | 117999  (92516-144527) | 154.62  (121.23-189.39) | -1.54 (-1.58 to -1.51) | <0.001 |
|  | 70-74 | 48138  (37792-60846) | 255.02  (200.2-322.34) | 80671  (62972-100250) | 132.13  (103.14-164.2) | -1.96 (-1.99 to -1.92) | <0.001 |
|  | 75-79 | 28030  (22274-34591) | 245.3  (194.92-302.72) | 42727  (33868-52127) | 116.14  (92.06-141.7) | -2.24 (-2.28 to -2.2) | <0.001 |
|  | 80-84 | 12829  (9978-16043) | 229.74  (178.69-287.29) | 23825  (18802-29832) | 106.06  (83.69-132.8) | -2.3 (-2.35 to -2.26) | <0.001 |
|  | 85-89 | 4163  (3323-5142) | 208.56  (166.46-257.58) | 12435  (10020-15257) | 99.7  (80.34-122.33) | -2.21 (-2.26 to -2.16) | <0.001 |
|  | 90-94 | 700  (559-851) | 185.82  (148.4-226.06) | 4300  (3459-5212) | 94.95  (76.37-115.08) | -2.02 (-2.06 to -1.98) | <0.001 |
|  | 95+ | 110  (84-144) | 168.14  (128.32-220.04) | 1163  (893-1546) | 93.66  (71.96-124.52) | -1.75 (-1.79 to -1.72) | <0.001 |
| **Deaths** | 15-19 | 3112  (2113-4434) | 2.45  (1.66-3.49) | 185  (117-266) | 0.23  (0.15-0.33) | -6.93 (-7.08 to -6.76) | <0.001 |
|  | 20-24 | 5606  (3651-7977) | 4.24  (2.76-6.03) | 285  (181-434) | 0.4  (0.26-0.61) | -6.83 (-6.98 to -6.67) | <0.001 |
|  | 25-29 | 4720  (3033-6632) | 4.28  (2.75-6.02) | 522  (346-796) | 0.67  (0.44-1.02) | -5.59 (-5.74 to -5.44) | <0.001 |
|  | 30-34 | 4155  (2832-5894) | 4.7  (3.2-6.66) | 887  (587-1368) | 0.83  (0.55-1.27) | -5.21 (-5.35 to -5.07) | <0.001 |
|  | 35-39 | 4006  (2735-5588) | 4.37  (2.99-6.1) | 1012  (646-1468) | 0.86  (0.55-1.24) | -4.86 (-5.02 to -4.69) | <0.001 |
|  | 40-44 | 2704  (1852-3872) | 4.02  (2.75-5.75) | 780  (501-1194) | 0.86  (0.55-1.31) | -4.6 (-4.75 to -4.44) | <0.001 |
|  | 45-49 | 1987  (1363-2798) | 3.84  (2.63-5.41) | 775  (479-1236) | 0.78  (0.48-1.24) | -4.82 (-4.97 to -4.67) | <0.001 |
|  | 50-54 | 1903  (1323-2635) | 3.98  (2.77-5.51) | 799  (491-1226) | 0.67  (0.41-1.04) | -5.28 (-5.46 to -5.1) | <0.001 |
|  | 55-59 | 1752  (1231-2436) | 4.03  (2.83-5.6) | 661  (443-985) | 0.56  (0.37-0.83) | -5.88 (-6.14 to -5.62) | <0.001 |
|  | 60-64 | 1690  (1202-2365) | 4.77  (3.39-6.67) | 522  (337-771) | 0.64  (0.41-0.95) | -5.91 (-6.13 to -5.65) | <0.001 |
|  | 65-69 | 1513  (1095-2167) | 5.52  (3.99-7.9) | 567  (374-811) | 0.74  (0.49-1.06) | -5.93 (-6.17 to -5.67) | <0.001 |
|  | 70-74 | 814  (573-1161) | 4.31  (3.03-6.15) | 382  (242-561) | 0.63  (0.4-0.92) | -5.67 (-5.93 to -5.44) | <0.001 |
|  | 75-79 | 548  (392-757) | 4.79  (3.43-6.63) | 311  (199-464) | 0.84  (0.54-1.26) | -5.23 (-5.4 to -5.04) | <0.001 |
|  | 80-84 | 244  (172-362) | 4.37  (3.08-6.48) | 244  (137-397) | 1.09  (0.61-1.77) | -4.19 (-4.37 to -4) | <0.001 |
|  | 85-89 | 85  (58-119) | 4.23  (2.93-5.95) | 159  (96-249) | 1.28  (0.77-2) | -3.41 (-3.71 to -3.12) | <0.001 |
|  | 90-94 | 17  (12-24) | 4.54  (3.12-6.45) | 76  (46-120) | 1.68  (1.01-2.64) | -3.04 (-3.26 to -2.8) | <0.001 |
|  | 95+ | 2  (1-3) | 3.33  (2.27-5.07) | 19  (11-31) | 1.52  (0.87-2.49) | -2.38 (-2.55 to -2.23) | <0.001 |
| **DALYs** | 10-14 | 624  (91-1529) | 0.61  (0.09-1.49) | 772  (104-1882) | 0.87  (0.12-2.12) | 1.1 (1.05 to 1.13) | <0.001 |
|  | 15-19 | 354048  (268279-453565) | 278.77  (211.24-357.13) | 64868  (45238-85220) | 80.52  (56.15-105.78) | -3.66 (-3.74 to -3.57) | <0.001 |
|  | 20-24 | 952487  (702626-1237289) | 719.56  (530.8-934.71) | 201862  (134257-267670) | 285.29  (189.75-378.3) | -2.76 (-2.81 to -2.69) | <0.001 |
|  | 25-29 | 787758  (584169-1004575) | 715.01  (530.22-911.81) | 244803  (172459-326122) | 313.89  (221.13-418.16) | -2.47 (-2.53 to -2.4) | <0.001 |
|  | 30-34 | 481497  (361137-622224) | 544.06  (408.06-703.08) | 236846  (166254-315967) | 220.49  (154.77-294.15) | -2.75 (-2.83 to -2.66) | <0.001 |
|  | 35-39 | 385766  (287057-497541) | 421.3  (313.5-543.38) | 192445  (140925-255981) | 162.79  (119.21-216.53) | -2.87 (-2.95 to -2.78) | <0.001 |
|  | 40-44 | 229718  (171309-295368) | 341.39  (254.59-438.96) | 117680  (89509-153835) | 129.39  (98.42-169.15) | -2.93 (-3.01 to -2.85) | <0.001 |
|  | 45-49 | 150983  (113701-198753) | 291.72  (219.68-384.01) | 104154  (79305-134079) | 104.42  (79.51-134.42) | -3.16 (-3.24 to -3.06) | <0.001 |
|  | 50-54 | 125581  (94417-160787) | 262.51  (197.37-336.11) | 101494  (73961-134235) | 85.72  (62.47-113.38) | -3.38 (-3.49 to -3.28) | <0.001 |
|  | 55-59 | 99034  (74874-124544) | 227.67  (172.13-286.32) | 82559  (57490-110106) | 69.83  (48.63-93.13) | -3.48 (-3.61 to -3.35) | <0.001 |
|  | 60-64 | 76163  (56732-98850) | 214.83  (160.02-278.83) | 50610  (34710-68923) | 62.15  (42.63-84.64) | -3.7 (-3.82 to -3.58) | <0.001 |
|  | 65-69 | 57166  (42331-74290) | 208.55  (154.43-271.02) | 42773  (29998-55580) | 56.05  (39.31-72.83) | -3.9 (-4.01 to -3.8) | <0.001 |
|  | 70-74 | 30627  (23429-39295) | 162.25  (124.11-208.17) | 28688  (20229-37898) | 46.99  (33.13-62.07) | -3.64 (-3.75 to -3.54) | <0.001 |
|  | 75-79 | 17229  (12942-21487) | 150.78  (113.26-188.04) | 16430  (11622-21436) | 44.66  (31.59-58.27) | -3.6 (-3.73 to -3.47) | <0.001 |
|  | 80-84 | 6822  (4905-8916) | 122.16  (87.83-159.66) | 9405  (6715-12435) | 41.87  (29.89-55.36) | -3.2 (-3.3 to -3.1) | <0.001 |
|  | 85-89 | 2014  (1501-2632) | 100.92  (75.18-131.84) | 4760  (3451-6242) | 38.17  (27.67-50.04) | -2.85 (-2.99 to -2.71) | <0.001 |
|  | 90-94 | 333  (246-428) | 88.43  (65.25-113.57) | 1679  (1210-2242) | 37.09  (26.72-49.52) | -2.6 (-2.72 to -2.47) | <0.001 |
|  | 95+ | 45  (33-58) | 68.97  (50.95-88.66) | 408  (301-552) | 32.83  (24.26-44.43) | -2.24 (-2.32 to -2.15) | <0.001 |

AAPC: average annual percentage change, ASR: age-standardized rate, DUDs: drug use disorders, DALYs: disability-adjusted life years.

Supplementary Table S2**.** Number of cases and ASR of DUDs incidence, prevalence, deaths and DALYs in India (1990 vs. 2023), stratified by age, with AAPC from 1990 to 2023.

| **Measure** | **Age group** | **1990** | | **2023** | | **AAPC (95%CI)** **1990–2023** | ***P*** |
| --- | --- | --- | --- | --- | --- | --- | --- |
|  |  | **Number (95%UI)** | **ASR (95%UI)** | **Number (95%UI)** | **ASR (95%UI)** |  |  |
| **Incidence** | 10-14 | 43565  (7005-95897) | 45.05  (7.24-99.16) | 26920  (5581-68485) | 20.58  (4.27-52.36) | -2.35 (-2.47 to -2.21) | <0.001 |
|  | 15-19 | 150795  (107200-216514) | 180  (127.96-258.44) | 223280  (155770-315001) | 166.72  (116.31-235.2) | -0.18 (-0.23 to -0.12) | <0.001 |
|  | 20-24 | 149751  (104804-215279) | 193.98  (135.76-278.86) | 301179  (218310-396711) | 228.73  (165.79-301.28) | 0.52 (0.48 to 0.56) | <0.001 |
|  | 25-29 | 131882  (88207-191770) | 192.4  (128.68-279.77) | 271303  (189296-382501) | 215.17  (150.13-303.36) | 0.35 (0.34 to 0.36) | <0.001 |
|  | 30-34 | 125307  (88946-166446) | 212.81  (151.06-282.68) | 275864  (199105-361866) | 233.87  (168.8-306.78) | 0.3 (0.29 to 0.3) | <0.001 |
|  | 35-39 | 125660  (84268-175184) | 238.84  (160.17-332.97) | 287347  (196257-401138) | 261.75  (178.77-365.4) | 0.28 (0.26 to 0.29) | <0.001 |
|  | 40-44 | 97429  (63009-137918) | 224.62  (145.27-317.97) | 236705  (153996-329618) | 245.69  (159.84-342.13) | 0.28 (0.27 to 0.29) | <0.001 |
|  | 45-49 | 59115  (42312-81648) | 164.1  (117.45-226.65) | 149187  (105290-204085) | 182.35  (128.7-249.45) | 0.34 (0.33 to 0.35) | <0.001 |
|  | 50-54 | 34528  (24463-48975) | 117.14  (82.99-166.15) | 91233  (64197-128229) | 129.48  (91.11-181.98) | 0.32 (0.3 to 0.33) | <0.001 |
|  | 55-59 | 18819  (14886-23775) | 75.02  (59.34-94.78) | 47396  (37611-59170) | 80.05  (63.53-99.94) | 0.21 (0.2 to 0.22) | <0.001 |
|  | 60-64 | 10145  (7604-12836) | 50.57  (37.9-63.98) | 26303  (19840-32585) | 52.09  (39.29-64.53) | 0.1 (0.09 to 0.1) | <0.001 |
|  | 65-69 | 5964  (4501-7182) | 43.96  (33.18-52.94) | 17969  (14182-21115) | 45.49  (35.9-53.45) | 0.11 (0.1 to 0.11) | <0.001 |
|  | 70-74 | 3426  (2596-4213) | 39.94  (30.26-49.12) | 12082  (9613-14673) | 41.75  (33.22-50.7) | 0.14 (0.13 to 0.14) | <0.001 |
|  | 75-79 | 1948  (1382-2456) | 38.29  (27.17-48.27) | 7470  (5627-9060) | 40.87  (30.79-49.57) | 0.21 (0.2 to 0.22) | <0.001 |
|  | 80-84 | 1077  (749-1351) | 38.28  (26.62-48.03) | 4185  (3155-5143) | 41.32  (31.15-50.78) | 0.24 (0.23 to 0.25) | <0.001 |
|  | 85-89 | 372  (284-452) | 39.78  (30.4-48.28) | 1938  (1552-2327) | 42.96  (34.4-51.57) | 0.24 (0.23 to 0.25) | <0.001 |
|  | 90-94 | 92  (70-115) | 41.41  (31.24-51.73) | 605  (465-744) | 44.94  (34.54-55.31) | 0.26 (0.25 to 0.27) | <0.001 |
|  | 95+ | 21  (13-28) | 43.56  (28.21-58.37) | 146  (94-194) | 47.65  (30.71-63.19) | 0.29 (0.28 to 0.3) | <0.001 |
| **prevalence** | 10-14 | 35942  (4603-80410) | 37.16  (4.76-83.14) | 21436  (3318-57765) | 16.39  (2.54-44.17) | -2.46 (-2.59 to -2.31) | <0.001 |
|  | 15-19 | 454128  (234595-772684) | 542.07  (280.02-922.32) | 506834  (304040-827072) | 378.44  (227.02-617.55) | -1.03 (-1.1 to -0.95) | <0.001 |
|  | 20-24 | 694457  (497087-933109) | 899.55  (643.89-1208.68) | 1191617  (864339-1607891) | 904.97  (656.42-1221.1) | 0.04 (-0.01 to 0.09) | 0.076 |
|  | 25-29 | 572100  (418766-792412) | 834.62  (610.93-1156.03) | 1161025  (849381-1595328) | 920.81  (673.65-1265.26) | 0.31 (0.26 to 0.36) | <0.001 |
|  | 30-34 | 403437  (288017-565891) | 685.18  (489.15-961.08) | 901579  (661295-1257822) | 764.33  (560.63-1066.35) | 0.37 (0.34 to 0.4) | <0.001 |
|  | 35-39 | 303543  (220031-440008) | 576.95  (418.21-836.32) | 707615  (526697-1022085) | 644.58  (479.78-931.03) | 0.36 (0.34 to 0.38) | <0.001 |
|  | 40-44 | 225773  (159536-319474) | 520.52  (367.81-736.54) | 543705  (387961-742462) | 564.34  (402.68-770.64) | 0.25 (0.24 to 0.27) | <0.001 |
|  | 45-49 | 159159  (112941-226227) | 441.81  (313.51-627.98) | 378533  (276135-527795) | 462.68  (337.52-645.13) | 0.16 (0.14 to 0.17) | <0.001 |
|  | 50-54 | 106199  (79178-136516) | 360.29  (268.62-463.14) | 256046  (193986-329016) | 363.38  (275.3-466.93) | 0.04 (0.02 to 0.06) | <0.001 |
|  | 55-59 | 69930  (53791-87292) | 278.78  (214.44-347.99) | 164630  (126570-202270) | 278.06  (213.78-341.64) | 0 (-0.01 to 0.02) | 0.498 |
|  | 60-64 | 42000  (33384-53056) | 209.35  (166.41-264.46) | 106531  (83967-132134) | 210.97  (166.28-261.67) | 0.03 (0.02 to 0.04) | <0.001 |
|  | 65-69 | 22197  (17625-27895) | 163.62  (129.91-205.62) | 64611  (51643-79982) | 163.56  (130.73-202.47) | 0 (0 to 0) | 0.715 |
|  | 70-74 | 11109  (8734-14432) | 129.52  (101.82-168.25) | 36402  (28649-45420) | 125.78  (98.99-156.94) | -0.09 (-0.1 to -0.08) | <0.001 |
|  | 75-79 | 5468  (4253-6877) | 107.46  (83.58-135.14) | 18701  (14803-23088) | 102.32  (80.99-126.33) | -0.14 (-0.15 to -0.13) | <0.001 |
|  | 80-84 | 2670  (2070-3495) | 94.9  (73.6-124.23) | 9134  (7108-11779) | 90.18  (70.18-116.3) | -0.15 (-0.16 to -0.13) | <0.001 |
|  | 85-89 | 850  (680-1061) | 90.81  (72.68-113.43) | 3990  (3265-4900) | 88.42  (72.37-108.6) | -0.06 (-0.08 to -0.05) | <0.001 |
|  | 90-94 | 206  (159-267) | 92.63  (71.36-120.13) | 1243  (978-1579) | 92.37  (72.67-117.34) | 0 (-0.02 to 0.02) | 0.863 |
|  | 95+ | 47  (34-61) | 99.06  (72.02-128.42) | 316  (236-409) | 103.19  (77.16-133.59) | 0.15 (0.12 to 0.17) | <0.001 |
| **Deaths** | 15-19 | 305  (197-441) | 0.36  (0.23-0.53) | 234  (143-345) | 0.17  (0.11-0.26) | -2.19 (-2.38 to -2.01) | <0.001 |
|  | 20-24 | 396  (248-623) | 0.51  (0.32-0.81) | 470  (290-752) | 0.36  (0.22-0.57) | -0.93 (-1.15 to -0.73) | <0.001 |
|  | 25-29 | 305  (196-443) | 0.44  (0.29-0.65) | 566  (365-873) | 0.45  (0.29-0.69) | 0.06 (-0.07 to 0.2) | 0.309 |
|  | 30-34 | 263  (168-374) | 0.45  (0.29-0.64) | 589  (391-875) | 0.5  (0.33-0.74) | 0.45 (0.27 to 0.64) | <0.001 |
|  | 35-39 | 230  (150-328) | 0.44  (0.28-0.62) | 543  (363-847) | 0.49  (0.33-0.77) | 0.54 (0.35 to 0.73) | <0.001 |
|  | 40-44 | 212  (136-309) | 0.49  (0.31-0.71) | 517  (349-773) | 0.54  (0.36-0.8) | 0.35 (0.2 to 0.51) | <0.001 |
|  | 45-49 | 247  (156-365) | 0.69  (0.43-1.01) | 557  (343-847) | 0.68  (0.42-1.04) | 0.12 (-0.1 to 0.34) | 0.206 |
|  | 50-54 | 188  (120-263) | 0.64  (0.41-0.89) | 442  (291-654) | 0.63  (0.41-0.93) | 0 (-0.15 to 0.15) | 0.986 |
|  | 55-59 | 266  (173-396) | 1.06  (0.69-1.58) | 642  (425-981) | 1.08  (0.72-1.66) | 0.12 (-0.05 to 0.3) | 0.159 |
|  | 60-64 | 208  (140-284) | 1.04  (0.7-1.41) | 447  (295-662) | 0.89  (0.58-1.31) | -0.43 (-0.67 to -0.18) | 0.002 |
|  | 65-69 | 197  (130-288) | 1.45  (0.96-2.12) | 458  (299-695) | 1.16  (0.76-1.76) | -0.64 (-0.82 to -0.49) | <0.001 |
|  | 70-74 | 103  (67-151) | 1.2  (0.78-1.75) | 282  (186-426) | 0.97  (0.64-1.47) | -0.61 (-0.73 to -0.49) | <0.001 |
|  | 75-79 | 66  (43-93) | 1.29  (0.84-1.83) | 212  (144-310) | 1.16  (0.79-1.7) | -0.31 (-0.47 to -0.14) | 0.001 |
|  | 80-84 | 37  (24-51) | 1.3  (0.87-1.8) | 134  (92-187) | 1.32  (0.91-1.84) | 0.09 (-0.07 to 0.22) | 0.208 |
|  | 85-89 | 15  (10-21) | 1.61  (1.06-2.29) | 92  (64-134) | 2.04  (1.43-2.97) | 0.85 (0.57 to 1.01) | <0.001 |
|  | 90-94 | 5  (3-7) | 2.3  (1.49-3.35) | 44  (30-68) | 3.26  (2.2-5.07) | 0.98 (0.84 to 1.13) | <0.001 |
|  | 95+ | 1  (1-2) | 2.98  (1.89-4.47) | 10  (7-16) | 3.28  (2.13-5.18) | 0.32 (0.17 to 0.48) | 0.001 |
| **DALYs** | 10-14 | 1077  (142-2388) | 1.11  (0.15-2.47) | 661  (100-1855) | 0.51  (0.08-1.42) | -2.38 (-2.51 to -2.23) | <0.001 |
|  | 15-19 | 51347  (36947-68611) | 61.29  (44.1-81.9) | 64475  (43562-84846) | 48.14  (32.53-63.35) | -0.7 (-0.76 to -0.63) | <0.001 |
|  | 20-24 | 110819  (80909-149315) | 143.55  (104.8-193.41) | 207642  (143454-275166) | 157.69  (108.95-208.97) | 0.35 (0.29 to 0.4) | <0.001 |
|  | 25-29 | 101221  (72808-131912) | 147.67  (106.22-192.44) | 225826  (160835-294998) | 179.1  (127.56-233.96) | 0.63 (0.57 to 0.69) | <0.001 |
|  | 30-34 | 70220  (50338-95754) | 119.26  (85.49-162.62) | 174808  (129402-231876) | 148.2  (109.7-196.58) | 0.69 (0.62 to 0.79) | <0.001 |
|  | 35-39 | 50320  (36006-69024) | 95.64  (68.44-131.2) | 130796  (97431-171569) | 119.14  (88.75-156.28) | 0.73 (0.65 to 0.82) | <0.001 |
|  | 40-44 | 36225  (26378-46518) | 83.52  (60.82-107.25) | 96957  (71700-128435) | 100.64  (74.42-133.31) | 0.61 (0.53 to 0.69) | <0.001 |
|  | 45-49 | 30200  (22028-39611) | 83.83  (61.15-109.96) | 77416  (58115-101415) | 94.63  (71.03-123.96) | 0.41 (0.33 to 0.49) | <0.001 |
|  | 50-54 | 22340  (16321-28992) | 75.79  (55.37-98.36) | 59192  (42893-77150) | 84  (60.87-109.49) | 0.34 (0.28 to 0.39) | <0.001 |
|  | 55-59 | 20933  (15753-28309) | 83.45  (62.8-112.86) | 52952  (37927-66858) | 89.44  (64.06-112.92) | 0.24 (0.17 to 0.31) | <0.001 |
|  | 60-64 | 14810  (10156-20332) | 73.82  (50.62-101.35) | 36234  (25846-47921) | 71.76  (51.18-94.9) | -0.09 (-0.18 to -0.01) | 0.024 |
|  | 65-69 | 10040  (7160-13600) | 74  (52.78-100.25) | 26534  (19453-35028) | 67.17  (49.25-88.67) | -0.26 (-0.34 to -0.19) | <0.001 |
|  | 70-74 | 4921  (3519-6510) | 57.37  (41.03-75.89) | 14897  (11023-18845) | 51.48  (38.09-65.12) | -0.3 (-0.36 to -0.24) | <0.001 |
|  | 75-79 | 2545  (1847-3212) | 50.01  (36.3-63.12) | 8456  (6421-10752) | 46.27  (35.14-58.83) | -0.24 (-0.28 to -0.19) | <0.001 |
|  | 80-84 | 1209  (861-1553) | 42.98  (30.6-55.21) | 4227  (3227-5324) | 41.73  (31.86-52.56) | -0.06 (-0.11 to -0.01) | 0.024 |
|  | 85-89 | 391  (285-504) | 41.85  (30.5-53.87) | 2047  (1583-2600) | 45.36  (35.08-57.63) | 0.27 (0.17 to 0.34) | <0.001 |
|  | 90-94 | 103  (76-132) | 46.1  (34.19-59.15) | 732  (552-972) | 54.39  (41.06-72.21) | 0.51 (0.4 to 0.63) | <0.001 |
|  | 95+ | 25  (19-32) | 51.83  (39.48-66.57) | 171  (127-226) | 55.73  (41.55-73.71) | 0.26 (0.2 to 0.33) | <0.001 |

AAPC: average annual percentage change, ASR: age-standardized rate, DUDs: drug use disorders, DALYs: disability-adjusted life years.

Supplementary Table S3**.** Number of cases and ASR of DUDs incidence, prevalence, deaths and DALYs in the United States (1990 vs. 2023), stratified by age, with AAPC from 1990 to 2023.

| **Measure** | **Age group** | **1990** | | **2023** | | **AAPC (95%CI)** **1990–2023** | ***P*** |
| --- | --- | --- | --- | --- | --- | --- | --- |
|  |  | **Number (95%UI)** | **ASR (95%UI)** | **Number (95%UI)** | **ASR (95%UI)** |  |  |
| **Incidence** | 10-14 | 89625  (47359-153042) | 508.87  (268.89-868.94) | 101249  (53967-172376) | 481.54  (256.66-819.82) | -0.16 (-0.18 to -0.13) | <0.001 |
|  | 15-19 | 213695  (163072-296411) | 1176.61  (897.87-1632.04) | 386043  (319880-480438) | 1748.81  (1449.08-2176.42) | 1.22 (1.2 to 1.24) | <0.001 |
|  | 20-24 | 112650  (85394-145460) | 574.24  (435.3-741.49) | 313967  (264837-375535) | 1444.9  (1218.81-1728.25) | 2.86 (2.81 to 2.91) | <0.001 |
|  | 25-29 | 121263  (87783-164324) | 557.62  (403.66-755.63) | 217445  (170938-279139) | 989.62  (777.96-1270.4) | 1.75 (1.73 to 1.77) | <0.001 |
|  | 30-34 | 131510  (98450-174344) | 588.89  (440.86-780.71) | 190734  (142089-251466) | 808.18  (602.06-1065.52) | 0.97 (0.95 to 0.98) | <0.001 |
|  | 35-39 | 126927  (86263-175440) | 622.95  (423.37-861.04) | 186422  (131590-255964) | 826.03  (583.07-1134.16) | 0.87 (0.85 to 0.89) | <0.001 |
|  | 40-44 | 99506  (63774-137996) | 550.74  (352.97-763.77) | 156618  (104704-223090) | 714.73  (477.82-1018.07) | 0.81 (0.79 to 0.83) | <0.001 |
|  | 45-49 | 51941  (36111-71365) | 371.06  (257.97-509.82) | 92662  (68917-119802) | 466.82  (347.19-603.54) | 0.71 (0.69 to 0.72) | <0.001 |
|  | 50-54 | 26280  (17088-37727) | 229.09  (148.96-328.87) | 57408  (40041-79989) | 276.44  (192.81-385.17) | 0.58 (0.57 to 0.6) | <0.001 |
|  | 55-59 | 11889  (8857-15866) | 113.12  (84.27-150.95) | 27330  (20934-35456) | 132.11  (101.19-171.4) | 0.49 (0.48 to 0.5) | <0.001 |
|  | 60-64 | 5410  (4027-6790) | 50.77  (37.8-63.72) | 11648  (8894-14835) | 54.65  (41.73-69.61) | 0.23 (0.22 to 0.24) | <0.001 |
|  | 65-69 | 4287  (3343-5085) | 42.54  (33.17-50.46) | 8618  (7071-10187) | 44.86  (36.8-53.02) | 0.17 (0.16 to 0.18) | <0.001 |
|  | 70-74 | 3067  (2322-3776) | 37.92  (28.72-46.7) | 6318  (5133-7653) | 40.72  (33.08-49.32) | 0.23 (0.21 to 0.24) | <0.001 |
|  | 75-79 | 2267  (1559-2909) | 36.97  (25.42-47.44) | 4792  (3432-6008) | 42.24  (30.25-52.95) | 0.42 (0.41 to 0.43) | <0.001 |
|  | 80-84 | 1436  (978-1797) | 36.46  (24.84-45.63) | 2992  (2162-3723) | 43.11  (31.15-53.63) | 0.52 (0.51 to 0.54) | <0.001 |
|  | 85-89 | 738  (556-916) | 36.27  (27.29-45.01) | 1650  (1287-2036) | 43.28  (33.75-53.4) | 0.55 (0.54 to 0.56) | <0.001 |
|  | 90-94 | 267  (189-334) | 36.93  (26.1-46.19) | 747  (541-954) | 43.43  (31.44-55.46) | 0.5 (0.49 to 0.51) | <0.001 |
|  | 95+ | 86  (53-113) | 38.43  (23.9-50.55) | 272  (170-359) | 43.29  (27.02-57.08) | 0.36 (0.35 to 0.37) | <0.001 |
| **prevalence** | 10-14 | 73438  (37742-126415) | 416.96  (214.29-717.75) | 82372  (42587-141032) | 391.76  (202.54-670.74) | -0.18 (-0.2 to -0.16) | <0.001 |
|  | 15-19 | 935515  (628677-1356060) | 5150.95  (3461.5-7466.47) | 1328902  (978553-1823698) | 6020.03  (4432.92-8261.5) | 0.48 (0.46 to 0.51) | <0.001 |
|  | 20-24 | 1136477  (922480-1390439) | 5793.24  (4702.38-7087.81) | 2226653  (1940512-2545155) | 10247.27  (8930.42-11713.04) | 1.73 (1.69 to 1.76) | <0.001 |
|  | 25-29 | 978126  (801077-1187140) | 4497.82  (3683.68-5458.96) | 2283707  (2050771-2585057) | 10393.45  (9333.33-11764.93) | 2.55 (2.5 to 2.6) | <0.001 |
|  | 30-34 | 697767  (577604-831385) | 3124.57  (2586.49-3722.91) | 1929870  (1685116-2206951) | 8177.28  (7140.21-9351.34) | 2.97 (2.93 to 3.01) | <0.001 |
|  | 35-39 | 471835  (393034-590649) | 2315.73  (1928.98-2898.86) | 1426410  (1232819-1666177) | 6320.35  (5462.56-7382.74) | 3.12 (3.07 to 3.15) | <0.001 |
|  | 40-44 | 319409  (263395-388920) | 1767.85  (1457.82-2152.57) | 1097779  (943772-1267214) | 5009.72  (4306.91-5782.94) | 3.23 (3.19 to 3.26) | <0.001 |
|  | 45-49 | 186119  (149358-232907) | 1329.61  (1066.99-1663.85) | 798774  (689328-925292) | 4024.08  (3472.71-4661.45) | 3.46 (3.42 to 3.49) | <0.001 |
|  | 50-54 | 113333  (90491-136366) | 987.93  (788.81-1188.71) | 662572  (560480-775754) | 3190.49  (2698.88-3735.49) | 3.66 (3.62 to 3.71) | <0.001 |
|  | 55-59 | 72806  (59590-87287) | 692.72  (566.97-830.49) | 445194  (378709-513670) | 2152.07  (1830.68-2483.08) | 3.54 (3.49 to 3.59) | <0.001 |
|  | 60-64 | 49310  (40845-59832) | 462.76  (383.32-561.52) | 259615  (219436-305824) | 1218.18  (1029.65-1435) | 3.02 (2.98 to 3.07) | <0.001 |
|  | 65-69 | 33158  (27555-40022) | 329.02  (273.43-397.13) | 130609  (108920-158501) | 679.79  (566.9-824.96) | 2.28 (2.25 to 2.32) | <0.001 |
|  | 70-74 | 19678  (16514-23605) | 243.34  (204.21-291.9) | 61563  (51726-73279) | 396.76  (333.36-472.27) | 1.51 (1.47 to 1.56) | <0.001 |
|  | 75-79 | 11913  (9888-14177) | 194.27  (161.24-231.19) | 31893  (26887-36984) | 281.12  (237-325.99) | 1.15 (1.13 to 1.18) | <0.001 |
|  | 80-84 | 6652  (5423-8257) | 168.95  (137.74-209.71) | 16997  (14184-20639) | 244.85  (204.32-297.31) | 1.16 (1.13 to 1.18) | <0.001 |
|  | 85-89 | 3153  (2605-3794) | 154.92  (127.96-186.41) | 8782  (7386-10366) | 230.33  (193.71-271.87) | 1.24 (1.21 to 1.27) | <0.001 |
|  | 90-94 | 1061  (867-1288) | 146.59  (119.79-177.82) | 3831  (3167-4574) | 222.59  (184.01-265.74) | 1.31 (1.28 to 1.33) | <0.001 |
|  | 95+ | 318  (255-388) | 142.59  (114.46-173.71) | 1317  (1034-1602) | 209.5  (164.54-254.81) | 1.19 (1.17 to 1.2) | <0.001 |
| **Deaths** | 15-19 | 100  (73-132) | 0.55  (0.4-0.73) | 1443  (1072-1831) | 6.54  (4.86-8.3) | 8.11 (7.9 to 8.33) | <0.001 |
|  | 20-24 | 366  (274-486) | 1.86  (1.4-2.48) | 4886  (3848-5954) | 22.48  (17.71-27.4) | 8.04 (7.74 to 8.34) | <0.001 |
|  | 25-29 | 787  (590-1027) | 3.62  (2.71-4.72) | 8648  (7154-10215) | 39.36  (32.56-46.49) | 7.66 (7.43 to 7.89) | <0.001 |
|  | 30-34 | 1157  (866-1477) | 5.18  (3.88-6.61) | 12446  (10183-14609) | 52.74  (43.15-61.9) | 7.42 (7.2 to 7.66) | <0.001 |
|  | 35-39 | 1174  (891-1520) | 5.76  (4.37-7.46) | 13195  (10827-15507) | 58.47  (47.97-68.71) | 7.38 (7.17 to 7.59) | <0.001 |
|  | 40-44 | 817  (611-1052) | 4.52  (3.38-5.82) | 13045  (10536-15539) | 59.53  (48.08-70.91) | 8.29 (8.11 to 8.49) | <0.001 |
|  | 45-49 | 432  (326-555) | 3.09  (2.33-3.97) | 10668  (8461-12892) | 53.74  (42.63-64.95) | 9.09 (8.97 to 9.2) | <0.001 |
|  | 50-54 | 228  (174-288) | 1.99  (1.52-2.51) | 10051  (7768-12491) | 48.4  (37.41-60.15) | 10.24 (10.09 to 10.39) | <0.001 |
|  | 55-59 | 160  (123-198) | 1.52  (1.17-1.88) | 8919  (6839-11281) | 43.11  (33.06-54.53) | 10.82 (10.7 to 10.95) | <0.001 |
|  | 60-64 | 123  (97-150) | 1.16  (0.91-1.41) | 6287  (4833-8055) | 29.5  (22.68-37.8) | 10.42 (10.28 to 10.55) | <0.001 |
|  | 65-69 | 110  (87-135) | 1.09  (0.86-1.34) | 3369  (2607-4312) | 17.54  (13.57-22.44) | 8.87 (8.77 to 8.96) | <0.001 |
|  | 70-74 | 86  (70-102) | 1.06  (0.86-1.26) | 1260  (1011-1579) | 8.12  (6.51-10.18) | 6.58 (6.46 to 6.71) | <0.001 |
|  | 75-79 | 75  (61-90) | 1.22  (0.99-1.47) | 508  (403-622) | 4.47  (3.55-5.48) | 4.2 (4.1 to 4.31) | <0.001 |
|  | 80-84 | 77  (60-93) | 1.95  (1.52-2.37) | 281  (222-348) | 4.05  (3.2-5.02) | 2.3 (2.21 to 2.39) | <0.001 |
|  | 85-89 | 56  (42-69) | 2.76  (2.06-3.41) | 179  (132-222) | 4.69  (3.46-5.83) | 1.71 (1.59 to 1.84) | <0.001 |
|  | 90-94 | 27  (20-34) | 3.72  (2.73-4.7) | 104  (74-133) | 6.03  (4.29-7.72) | 1.41 (1.34 to 1.48) | <0.001 |
|  | 95+ | 9  (6-11) | 3.97  (2.79-5.09) | 46  (29-61) | 7.3  (4.69-9.63) | 1.84 (1.76 to 1.94) | <0.001 |
| **DALYs** | 10-14 | 2657  (1276-4265) | 15.09  (7.25-24.22) | 3172  (1564-5132) | 15.09  (7.44-24.41) | 0.02 (-0.01 to 0.04) | 0.152 |
|  | 15-19 | 67162  (44705-90106) | 369.8  (246.15-496.13) | 261793  (205444-317274) | 1185.94  (930.68-1437.28) | 3.61 (3.54 to 3.69) | <0.001 |
|  | 20-24 | 151577  (109581-188670) | 772.67  (558.59-961.75) | 853785  (678911-1034747) | 3929.2  (3124.41-4762) | 5.06 (4.97 to 5.15) | <0.001 |
|  | 25-29 | 193783  (145269-235955) | 891.09  (668.01-1085.02) | 1188363  (975597-1395691) | 5408.4  (4440.07-6351.97) | 5.69 (5.59 to 5.77) | <0.001 |
|  | 30-34 | 181903  (139780-219504) | 814.55  (625.93-982.93) | 1291320  (1084173-1512088) | 5471.61  (4593.88-6407.05) | 5.98 (5.84 to 6.11) | <0.001 |
|  | 35-39 | 142589  (110934-175141) | 699.82  (544.45-859.58) | 1122868  (945580-1318741) | 4975.37  (4189.82-5843.28) | 6.18 (6.09 to 6.29) | <0.001 |
|  | 40-44 | 93828  (74026-115535) | 519.32  (409.72-639.46) | 950409  (805527-1108249) | 4337.2  (3676.03-5057.5) | 6.7 (6.62 to 6.79) | <0.001 |
|  | 45-49 | 50612  (40486-61724) | 361.57  (289.23-440.95) | 694567  (586089-818232) | 3499.1  (2952.61-4122.1) | 7.2 (7.13 to 7.27) | <0.001 |
|  | 50-54 | 28399  (21595-35650) | 247.56  (188.25-310.77) | 576665  (482197-691685) | 2776.82  (2321.92-3330.67) | 7.63 (7.57 to 7.7) | <0.001 |
|  | 55-59 | 18122  (13588-22667) | 172.42  (129.29-215.66) | 423134  (349477-515648) | 2045.43  (1689.37-2492.64) | 7.85 (7.77 to 7.93) | <0.001 |
|  | 60-64 | 12337  (9020-15961) | 115.78  (84.65-149.79) | 250221  (206791-308743) | 1174.1  (970.32-1448.7) | 7.39 (7.29 to 7.48) | <0.001 |
|  | 65-69 | 8475  (6389-10788) | 84.09  (63.39-107.04) | 113619  (93182-143053) | 591.36  (484.99-744.56) | 6.17 (6.09 to 6.25) | <0.001 |
|  | 70-74 | 5113  (3836-6537) | 63.23  (47.43-80.84) | 38568  (31828-46808) | 248.56  (205.13-301.67) | 4.33 (4.26 to 4.4) | <0.001 |
|  | 75-79 | 3302  (2552-4150) | 53.84  (41.61-67.67) | 14746  (12212-17957) | 129.98  (107.64-158.28) | 2.78 (2.72 to 2.85) | <0.001 |
|  | 80-84 | 2182  (1689-2686) | 55.43  (42.89-68.21) | 7115  (5708-8670) | 102.49  (82.22-124.89) | 1.93 (1.88 to 1.99) | <0.001 |
|  | 85-89 | 1156  (903-1399) | 56.81  (44.34-68.72) | 3677  (2967-4440) | 96.45  (77.81-116.45) | 1.67 (1.62 to 1.72) | <0.001 |
|  | 90-94 | 439  (342-542) | 60.62  (47.29-74.91) | 1750  (1367-2112) | 101.7  (79.44-122.72) | 1.57 (1.54 to 1.6) | <0.001 |
|  | 95+ | 135  (104-163) | 60.71  (46.73-73.27) | 667  (515-818) | 106.1  (81.88-130.18) | 1.71 (1.67 to 1.76) | <0.001 |

AAPC: average annual percentage change, ASR: age-standardized rate, DUDs: drug use disorders, DALYs: disability-adjusted life years.

Supplementary Table S4**.** Number of cases and ASR of DUDs incidence, prevalence, deaths and DALYs in 1990 and 2023, stratified by sex, with AAPC from 1990 to 2023.

| **laction** | **Measure** | **Sex** | **1990** | | **2023** | | **AAPC (95%CI)**  **1990–2023** | ***P*** |
| --- | --- | --- | --- | --- | --- | --- | --- | --- |
|  |  |  | **Number (95%UI)** | **ASR (95%UI)** | **Number (95%UI)** | **ASR (95%UI)** |  |  |
| **China** | Incidence | Male | 1649352  (1402198-1942017) | 238.64  (204.72-280.4) | 1489409  (1250103-1790135) | 206.56  (175.32-246.13) | -0.43 (-0.44 to -0.42) | <0.001 |
|  |  | Female | 1483153  (1252792-1764035) | 230.77  (196.24-273.82) | 1230836  (1015197-1517172) | 178.22  (149.08-217.06) | -0.78 (-0.79 to -0.76) | <0.001 |
|  | prevalence | Male | 5992681  (5015500-7290507) | 827.59  (698.83-991.34) | 4229697  (3582215-5072075) | 645.68  (542.61-789.89) | -0.7 (-0.74 to -0.66) | <0.001 |
|  |  | Female | 5142259  (4361858-6176774) | 763.94  (657.03-902.86) | 3119177  (2659210-3635664) | 496.67  (409.99-590.09) | -1.27 (-1.29 to -1.23) | <0.001 |
|  | Deaths | Male | 20206  (12582-31625) | 3.45  (2.17-5.39) | 6406  (3629-9594) | 0.73  (0.42-1.12) | -4.56 (-4.71 to -4.4) | <0.001 |
|  |  | Female | 14652  (8806-23576) | 2.45  (1.47-3.92) | 1780  (1054-2960) | 0.21  (0.12-0.35) | -7.25 (-7.44 to -7.06) | <0.001 |
|  | DALYs | Male | 2008434  (1433110-2658505) | 297.88  (214.54-395.11) | 886050  (681877-1117381) | 123.48  (92.9-156.37) | -2.68 (-2.75 to -2.58) | <0.001 |
|  |  | Female | 1749462  (1309564-2304062) | 270.5  (203.36-355.54) | 616187  (443135-777367) | 90.09  (64.28-114.99) | -3.25 (-3.33 to -3.16) | <0.001 |
| **India** | Incidence | Male | 554742  (454537-685694) | 128.24  (106.24-156.6) | 1095198  (916008-1289194) | 134.94  (113.5-158.39) | 0.19 (0.17 to 0.22) | <0.001 |
|  |  | Female | 405154  (336057-500678) | 106.77  (89.3-131.04) | 885914  (741156-1078883) | 115.49  (96.98-140.76) | 0.24 (0.22 to 0.26) | <0.001 |
|  | prevalence | Male | 2090927  (1648767-2717128) | 482.32  (387.3-609.36) | 3801540  (3026940-4506140) | 466.52  (372.81-550.5) | -0.06 (-0.09 to -0.02) | 0.001 |
|  |  | Female | 1018289  (826001-1220454) | 261.72  (216.24-308.54) | 2272408  (1845663-2684792) | 293.68  (239.81-346.5) | 0.38 (0.34 to 0.41) | <0.001 |
|  | Deaths | Male | 1753  (1003-2980) | 0.53  (0.3-0.91) | 3530  (2130-5983) | 0.5  (0.3-0.84) | -0.15 (-0.4 to 0.09) | 0.226 |
|  |  | Female | 1289  (761-2038) | 0.41  (0.25-0.65) | 2708  (1719-3945) | 0.38  (0.24-0.55) | -0.27 (-0.39 to -0.12) | <0.001 |
|  | DALYs | Male | 304656  (221415-401665) | 74.63  (54.1-98.02) | 671700  (496924-831896) | 83.89  (62.28-103.39) | 0.42 (0.31 to 0.53) | <0.001 |
|  |  | Female | 224091  (165387-282312) | 60.48  (44.61-76.38) | 512323  (390458-643460) | 67.21  (51.37-84.32) | 0.33 (0.27 to 0.39) | <0.001 |
| **the United States** | Incidence | Male | 544159  (451239-651135) | 428.23  (353.34-518.44) | 954488  (840645-1088927) | 633.08  (554.73-726.78) | 1.2 (1.18 to 1.22) | <0.001 |
|  |  | Female | 458685  (373703-557163) | 353.32  (288.24-426.33) | 812428  (708979-951758) | 537.44  (465.83-627.22) | 1.29 (1.27 to 1.31) | <0.001 |
|  | prevalence | Male | 3157105  (2646736-3687923) | 2392.63  (1986.82-2849.35) | 7263628  (6579036-8040898) | 4559.44  (4090.63-5089.32) | 1.98 (1.96 to 2.01) | <0.001 |
|  |  | Female | 1952963  (1634197-2274913) | 1487.66  (1239.3-1759.91) | 5533211  (4970098-6108340) | 3453.06  (3096.39-3857.46) | 2.63 (2.6 to 2.68) | <0.001 |
|  | Deaths | Male | 4226  (3055-5763) | 3.01  (2.17-4.11) | 67001  (50555-86776) | 36.48  (27.74-46.98) | 7.89 (7.65 to 8.12) | <0.001 |
|  |  | Female | 1559  (1150-2053) | 1.04  (0.77-1.38) | 28332  (22002-35948) | 15.35  (11.95-19.39) | 8.63 (8.5 to 8.77) | <0.001 |
|  | DALYs | Male | 613569  (471872-732285) | 444.67  (341.33-531.16) | 4964007  (4015155-5948149) | 2927.25  (2377.01-3489.52) | 5.92 (5.81 to 6.01) | <0.001 |
|  |  | Female | 350205  (264920-425145) | 253.19  (191.41-307.93) | 2832432  (2321943-3398345) | 1684.65  (1375.08-2019.54) | 5.94 (5.84 to 6.07) | <0.001 |

AAPC: average annual percentage change, ASR: age-standardized rate, DUDs: drug use disorders, DALYs: disability-adjusted life years.

Supplementary Table S5**.** Number of cases and ASR of DUDs incidence, prevalence, deaths and DALYs in 2023, stratified by drug subtypes, with AAPC from 1990 to 2023.

| **Cause Name** | **Measure** | **Laction** | **Number in 2023**  **(95%UI)** | **ASR in 2023 (95%UI)** | **AAPC (95%CI)**  **1990-2023** | ***P*** |
| --- | --- | --- | --- | --- | --- | --- |
| **Amphetamine use disorders** | Incidence | China | 342001  (240114-463118) | 32.29  (21.67-45.21) | -1.38 (-1.41 to -1.35) | <0.001 |
|  |  | India | 27752  (19209-38622) | 1.75  (1.22-2.42) | 0.12 (0.12 to 0.13) | <0.001 |
|  |  | the United States | 128681  (92451-171711) | 44.48  (31.55-59.88) | 1.03 (0.94 to 1.12) | <0.001 |
|  | Prevalence | China | 3125257  (2307331-4105365) | 272.68  (198.84-367.2) | -1.4 (-1.44 to -1.36) | <0.001 |
|  |  | India | 186726  (130125-254823) | 11.7  (8.2-15.87) | 0.15 (0.14 to 0.15) | <0.001 |
|  |  | the United States | 1087645  (806516-1412557) | 361.16  (264.47-476.89) | 1.4 (1.27 to 1.53) | <0.001 |
|  | Deaths | China | 1856  (1014-3077) | 0.12  (0.07-0.2) | -2.79 (-2.97 to -2.54) | <0.001 |
|  |  | India | 254  (115-515) | 0.02  (0.01-0.03) | 2.27 (2.13 to 2.43) | <0.001 |
|  |  | the United States | 6593  (4309-9708) | 1.74  (1.15-2.55) | 10.52 (10.23 to 10.85) | <0.001 |
|  | DALYs | China | 493894  (318217-707511) | 41.91  (26.79-60.47) | -1.67 (-1.72 to -1.61) | <0.001 |
|  |  | India | 37811  (23745-54298) | 2.39  (1.51-3.44) | 0.62 (0.57 to 0.66) | <0.001 |
|  |  | the United States | 436677  (314045-563154) | 129.37  (93.42-167.12) | 4.26 (4.14 to 4.42) | <0.001 |
| **Cannabis use disorders** | Incidence | China | 367406  (270596-480329) | 31.29  (23.01-42.63) | 0.6 (0.58 to 0.62) | <0.001 |
|  |  | India | 656959  (478896-861034) | 40.72  (29.85-52.94) | -0.15 (-0.17 to -0.12) | <0.001 |
|  |  | the United States | 388407  (291480-559169) | 142.91  (106.47-211.95) | -0.03 (-0.04 to -0.02) | <0.001 |
|  | Prevalence | China | 2319466  (1724437-2986797) | 180.75  (128.92-235.04) | 0.66 (0.64 to 0.68) | <0.001 |
|  |  | India | 3856930  (2763413-4914076) | 240.95  (173.37-306.42) | -0.22 (-0.26 to -0.18) | <0.001 |
|  |  | the United States | 2905220  (2150513-3780203) | 973.43  (706.03-1274.08) | -0.01 (-0.02 to 0) | 0.004 |
|  | Deaths | China | — | — | — | — |
|  |  | India | — | — | — | — |
|  |  | the United States | — | — | — | — |
|  | DALYs | China | 67527  (41277-106068) | 5.27  (3.14-8.48) | 0.67 (0.64 to 0.69) | <0.001 |
|  |  | India | 111163  (66401-176837) | 6.94  (4.17-10.95) | -0.22 (-0.25 to -0.19) | <0.001 |
|  |  | the United States | 83108  (49098-129359) | 27.91  (16.15-43.45) | -0.03 (-0.03 to -0.02) | <0.001 |
| **Cocaine use disorders** | Incidence | China | 6224  (3614-8890) | 0.56  (0.32-0.83) | -0.49 (-0.53 to -0.45) | <0.001 |
|  |  | India | 8381  (5363-11943) | 0.53  (0.34-0.75) | -0.14 (-0.15 to -0.12) | <0.001 |
|  |  | the United States | 77269  (55182-106339) | 29.06  (20.68-40.1) | 0.71 (0.65 to 0.77) | <0.001 |
|  | Prevalence | China | 60443  (37501-90350) | 4.72  (2.89-7.17) | -0.54 (-0.59 to -0.5) | <0.001 |
|  |  | India | 72353  (49282-102565) | 4.67  (3.27-6.55) | -0.13 (-0.14 to -0.11) | <0.001 |
|  |  | the United States | 1916633  (1567838-2338803) | 598.59  (483.25-739.83) | 1.19 (1.13 to 1.24) | <0.001 |
|  | Deaths | China | 115  (44-197) | 0.01  (0-0.01) | -5.41 (-5.58 to -5.23) | <0.001 |
|  |  | India | 735  (384-1319) | 0.05  (0.03-0.09) | -0.61 (-0.74 to -0.48) | <0.001 |
|  |  | the United States | 10503  (7621-14133) | 2.72  (1.97-3.67) | 7.7 (7.5 to 7.89) | <0.001 |
|  | DALYs | China | 13982  (8268-21297) | 1.02  (0.59-1.61) | -3.09 (-3.17 to -3) | <0.001 |
|  |  | India | 42852  (25918-66118) | 2.8  (1.7-4.35) | -0.62 (-0.71 to -0.52) | <0.001 |
|  |  | the United States | 718284  (546765-907423) | 207.13  (157.96-260.5) | 3.49 (3.4 to 3.58) | <0.001 |
| **Opioid use disorders** | Incidence | China | 248076  (205892-290175) | 17.66  (14.51-21.16) | -1.65 (-1.7 to -1.59) | <0.001 |
|  |  | India | 326554  (266683-400342) | 20.6  (16.91-25.04) | 0.73 (0.68 to 0.79) | <0.001 |
|  |  | the United States | 447210  (376717-545902) | 156.63  (131.8-192.77) | 5.27 (5.17 to 5.39) | <0.001 |
|  | Prevalence | China | 1563297  (1297368-1827851) | 101.07  (81.88-119.49) | -1.92 (-1.98 to -1.86) | <0.001 |
|  |  | India | 1831332  (1473455-2218565) | 116.83  (95.08-140.03) | 0.76 (0.71 to 0.81) | <0.001 |
|  |  | the United States | 6990364  (6058906-7956232) | 2127.06  (1840.76-2446.11) | 5.98 (5.92 to 6.03) | <0.001 |
|  | Deaths | China | 4206  (2709-6179) | 0.23  (0.14-0.35) | -5.94 (-6.1 to -5.79) | <0.001 |
|  |  | India | 4208  (2835-6587) | 0.3  (0.2-0.46) | 0.01 (-0.2 to 0.22) | 0.832 |
|  |  | the United States | 72697  (57750-89669) | 19.98  (15.91-24.55) | 8.21 (8.01 to 8.44) | <0.001 |
|  | DALYs | China | 805983  (584521-1037403) | 51.67  (37.33-66.53) | -3.35 (-3.44 to -3.25) | <0.001 |
|  |  | India | 932998  (675485-1186452) | 59.75  (43.56-75.57) | 0.56 (0.49 to 0.64) | <0.001 |
|  |  | the United States | 6274116  (5192624-7430623) | 1865.45  (1541.23-2211.01) | 7.01 (6.93 to 7.09) | <0.001 |
| **Other drug use disorders** | Incidence | China | 1756539  (1356088-2240648) | 111.44  (85.36-145.41) | -0.38 (-0.39 to -0.37) | <0.001 |
|  |  | India | 961466  (716840-1222055) | 61.98  (46.52-78.41) | 0.32 (0.31 to 0.33) | <0.001 |
|  |  | the United States | 725348  (558370-949557) | 213.42  (162.04-283.19) | 0.82 (0.81 to 0.83) | <0.001 |
|  | Prevalence | China | 304864  (238102-392462) | 18.95  (14.6-24.83) | -0.66 (-0.68 to -0.64) | <0.001 |
|  |  | India | 137158  (101674-180986) | 8.91  (6.64-11.68) | 0.42 (0.41 to 0.43) | <0.001 |
|  |  | the United States | 203086  (167995-243830) | 58.19  (47.56-70.03) | 1.58 (1.56 to 1.61) | <0.001 |
|  | Deaths | China | 2008  (1120-3343) | 0.12  (0.07-0.2) | -5.94 (-6.16 to -5.73) | <0.001 |
|  |  | India | 1041  (581-1804) | 0.08  (0.04-0.13) | -0.47 (-0.58 to -0.37) | <0.001 |
|  |  | the United States | 5540  (4101-7368) | 1.53  (1.12-2.03) | 6.3 (6.09 to 6.55) | <0.001 |
|  | DALYs | China | 120851  (82836-179611) | 7.74  (5.25-11.45) | -5.42 (-5.59 to -5.23) | <0.001 |
|  |  | India | 59200  (37317-92814) | 3.91  (2.5-6.09) | -0.46 (-0.54 to -0.38) | <0.001 |
|  |  | the United States | 284254  (216149-372806) | 82.67  (62.61-108.09) | 5.5 (5.31 to 5.69) | <0.001 |

AAPC: average annual percentage change, ASR: age-standardized rate, DUDs: drug use disorders, DALYs: disability-adjusted life years.

Supplementary Table S6**.** Projected case number and age-standardized rate (ASR) of incidence for drug use disorders to 2040.

| **Year** | **China** | | **India** | | **the United States** | |
| --- | --- | --- | --- | --- | --- | --- |
|  | **Number** | **ASR** | **Number** | **ASR** | **Number** | **ASR** |
| 1994 | 3290859 | 294.086749 | 1050464 | 147.276063 | 1055050 | 494.115789 |
| 1995 | 3302155 | 293.708587 | 1075283 | 147.229815 | 1069750 | 495.436787 |
| 1996 | 3303193 | 293.028218 | 1100553 | 147.214085 | 1083799 | 496.187966 |
| 1997 | 3300886 | 292.109121 | 1127578 | 147.290537 | 1094917 | 496.357258 |
| 1998 | 3301881 | 290.992022 | 1155900 | 147.402153 | 1105267 | 496.604406 |
| 1999 | 3306088 | 289.726928 | 1185025 | 147.492446 | 1116941 | 497.591337 |
| 2000 | 3319552 | 288.36539 | 1214496 | 147.504732 | 1129978 | 499.975049 |
| 2001 | 3332349 | 286.429911 | 1241409 | 147.254786 | 1149094 | 503.609134 |
| 2002 | 3340643 | 283.614371 | 1265296 | 146.685019 | 1166798 | 507.670481 |
| 2003 | 3346950 | 280.241742 | 1287233 | 145.930913 | 1182986 | 512.027715 |
| 2004 | 3349857 | 276.624047 | 1308422 | 145.127874 | 1201039 | 516.584639 |
| 2005 | 3352402 | 273.071922 | 1330291 | 144.41122 | 1218274 | 521.263752 |
| 2006 | 3329144 | 268.04017 | 1352810 | 143.835939 | 1238661 | 527.243425 |
| 2007 | 3271052 | 260.846445 | 1376275 | 143.38343 | 1262123 | 535.35681 |
| 2008 | 3196799 | 253.083614 | 1401168 | 143.079824 | 1286925 | 544.524782 |
| 2009 | 3125311 | 246.338459 | 1427948 | 142.951734 | 1310662 | 553.733713 |
| 2010 | 3077873 | 242.210059 | 1457071 | 143.025583 | 1331004 | 561.988758 |
| 2011 | 3042189 | 239.883919 | 1494657 | 143.88837 | 1355195 | 571.522138 |
| 2012 | 2998351 | 237.576943 | 1543498 | 145.734876 | 1387579 | 584.16997 |
| 2013 | 2950585 | 235.452976 | 1597658 | 148.002988 | 1424539 | 598.511278 |
| 2014 | 2903146 | 233.673778 | 1650936 | 150.132789 | 1463065 | 613.028992 |
| 2015 | 2859792 | 232.402125 | 1696756 | 151.567696 | 1498794 | 626.303731 |
| 2016 | 2824766 | 232.205324 | 1735126 | 152.353586 | 1537697 | 640.978096 |
| 2017 | 2797524 | 233.159612 | 1771764 | 152.983335 | 1583285 | 658.973033 |
| 2018 | 2774993 | 234.682392 | 1807954 | 153.570394 | 1629089 | 677.820594 |
| 2019 | 2755134 | 236.185841 | 1845016 | 154.224635 | 1669748 | 695.006101 |
| 2020 | 2735449 | 237.080878 | 1883746 | 155.055632 | 1699420 | 708.137445 |
| 2021 | 2738175 | 239.199848 | 1932981 | 156.820053 | 1731083 | 721.773243 |
| 2022 | 2743613 | 241.265804 | 1977794 | 158.220657 | 1759299 | 730.624044 |
| 2023 | 2720246 | 240.642481 | 1981112 | 156.380926 | 1766916 | 730.388565 |
| 2024 | 2836594 | 244.472691 | 2027290 | 156.747722 | 1824561 | 747.391012 |
| 2025 | 2827524 | 246.375805 | 2054758 | 156.918003 | 1856060 | 757.378701 |
| 2026 | 2818560 | 248.275091 | 2081742 | 157.088451 | 1887841 | 767.366301 |
| 2027 | 2810535 | 250.455819 | 2104861 | 157.072828 | 1918598 | 777.58069 |
| 2028 | 2799625 | 252.633113 | 2127690 | 157.055287 | 1949197 | 787.7938 |
| 2029 | 2786959 | 254.805979 | 2150141 | 157.037294 | 1979490 | 798.004102 |
| 2030 | 2774577 | 256.976935 | 2171628 | 157.019393 | 2009903 | 808.212286 |
| 2031 | 2764663 | 259.147942 | 2191715 | 157.001274 | 2040834 | 818.419334 |
| 2032 | 2759557 | 261.126451 | 2206510 | 156.775745 | 2066151 | 826.151128 |
| 2033 | 2746470 | 263.107211 | 2220423 | 156.548018 | 2091818 | 833.88164 |
| 2034 | 2730082 | 265.088039 | 2233619 | 156.319837 | 2117625 | 841.609409 |
| 2035 | 2712499 | 267.06956 | 2245737 | 156.091863 | 2143586 | 849.335224 |
| 2036 | 2695787 | 269.051988 | 2256539 | 155.863983 | 2169572 | 857.060584 |
| 2037 | 2682325 | 270.875264 | 2259759 | 155.26088 | 2185991 | 861.13458 |
| 2038 | 2665039 | 272.700682 | 2261733 | 154.656273 | 2202093 | 865.209019 |
| 2039 | 2645848 | 274.527438 | 2262492 | 154.051941 | 2217749 | 869.283762 |
| 2040 | 2625248 | 276.354395 | 2261948 | 153.448439 | 2233057 | 873.358496 |

Supplementary Table S7**.** Projected case number and age-standardized rate (ASR) of prevalence for drug use disorders to 2040.

| **Year** | **China** | | **India** | | **the United States** | |
| --- | --- | --- | --- | --- | --- | --- |
|  | **Number** | **ASR** | **Number** | **ASR** | **Number** | **ASR** |
| 1994 | 12115087 | 1027.273822 | 3416138 | 474.104375 | 5113781 | 2390.226278 |
| 1995 | 12100998 | 1025.983103 | 3494754 | 474.316103 | 5137788 | 2389.558429 |
| 1996 | 11977961 | 1019.916183 | 3578618 | 474.933617 | 5167852 | 2387.802729 |
| 1997 | 11782516 | 1010.099489 | 3672292 | 476.459429 | 5197782 | 2383.727658 |
| 1998 | 11556217 | 997.791643 | 3771608 | 478.270641 | 5237428 | 2382.285411 |
| 1999 | 11328237 | 984.233197 | 3872497 | 479.743141 | 5294325 | 2388.429946 |
| 2000 | 11122789 | 970.746839 | 3970904 | 480.259158 | 5372850 | 2407.014765 |
| 2001 | 10879873 | 952.792401 | 4055208 | 478.846719 | 5501523 | 2437.296418 |
| 2002 | 10566985 | 927.041027 | 4122935 | 475.479212 | 5634661 | 2472.179382 |
| 2003 | 10237415 | 896.680236 | 4179908 | 470.995271 | 5768443 | 2510.34942 |
| 2004 | 9933883 | 864.915955 | 4232975 | 466.22779 | 5916928 | 2550.864905 |
| 2005 | 9686503 | 834.949223 | 4289786 | 462.003814 | 6066494 | 2592.596047 |
| 2006 | 9390079 | 799.693027 | 4357660 | 459.193266 | 6240224 | 2641.629861 |
| 2007 | 8985594 | 755.808081 | 4438096 | 457.787316 | 6441208 | 2703.781661 |
| 2008 | 8564047 | 711.973757 | 4527204 | 457.320628 | 6663015 | 2774.691247 |
| 2009 | 8222807 | 676.827155 | 4620861 | 457.325305 | 6894101 | 2849.985798 |
| 2010 | 8065172 | 659.002823 | 4714516 | 457.332054 | 7121482 | 2925.64421 |
| 2011 | 8021940 | 653.351864 | 4833831 | 459.824669 | 7394246 | 3017.129967 |
| 2012 | 7960595 | 648.974466 | 4994927 | 466.068567 | 7741100 | 3136.38301 |
| 2013 | 7879858 | 645.854709 | 5174452 | 473.772871 | 8140059 | 3275.86882 |
| 2014 | 7780154 | 643.961118 | 5347206 | 480.647964 | 8575634 | 3427.133243 |
| 2015 | 7668099 | 643.255144 | 5486115 | 484.404644 | 9019905 | 3582.627817 |
| 2016 | 7599487 | 648.53375 | 5578860 | 484.37092 | 9585800 | 3786.895314 |
| 2017 | 7593135 | 661.57347 | 5644986 | 482.344028 | 10308643 | 4056.762987 |
| 2018 | 7601534 | 677.769568 | 5702035 | 479.730132 | 11061261 | 4343.962513 |
| 2019 | 7584491 | 692.482494 | 5768196 | 477.931758 | 11725754 | 4600.34218 |
| 2020 | 7512648 | 701.050461 | 5860525 | 478.354014 | 12173182 | 4779.216158 |
| 2021 | 7501142 | 711.883001 | 6012494 | 483.846218 | 12496729 | 4914.321585 |
| 2022 | 7488195 | 720.884508 | 6150912 | 488.273535 | 12723615 | 4992.249121 |
| 2023 | 7348874 | 717.312867 | 6073948 | 476.234045 | 12796839 | 5001.727978 |
| 2024 | 7899905 | 749.49119 | 6182239 | 475.24961 | 14026394 | 5366.036387 |
| 2025 | 7911321 | 763.568703 | 6226693 | 473.211042 | 14567817 | 5535.486469 |
| 2026 | 7929326 | 777.644022 | 6268725 | 471.161621 | 15111550 | 5704.929772 |
| 2027 | 7959975 | 791.849247 | 6304808 | 468.947364 | 15573560 | 5845.127652 |
| 2028 | 7996681 | 806.023893 | 6338757 | 466.723656 | 16034426 | 5985.301485 |
| 2029 | 8037270 | 820.181118 | 6370606 | 464.492774 | 16493520 | 6125.43703 |
| 2030 | 8085986 | 834.32144 | 6398935 | 462.256136 | 16953648 | 6265.537 |
| 2031 | 8148112 | 848.443076 | 6422636 | 460.014213 | 17417665 | 6405.604719 |
| 2032 | 8208337 | 859.163062 | 6443289 | 458.119755 | 17763754 | 6500.56728 |
| 2033 | 8254172 | 869.848536 | 6461055 | 456.218584 | 18111546 | 6595.491223 |
| 2034 | 8299171 | 880.495946 | 6476515 | 454.313609 | 18460341 | 6690.356994 |
| 2035 | 8350130 | 891.120872 | 6487990 | 452.405223 | 18811971 | 6785.17322 |
| 2036 | 8414387 | 901.737984 | 6494074 | 450.492979 | 19167300 | 6879.951562 |
| 2037 | 8475636 | 908.130167 | 6492926 | 448.758128 | 19376980 | 6923.808023 |
| 2038 | 8495793 | 914.524048 | 6487957 | 447.018775 | 19587586 | 6967.63323 |
| 2039 | 8502975 | 920.904095 | 6479854 | 445.278227 | 19797927 | 7011.402675 |
| 2040 | 8497111 | 927.278959 | 6468249 | 443.536477 | 20008911 | 7055.123769 |

Supplementary Table S8**.** Projected case number and age-standardized rate (ASR) of deaths for drug use disorders to 2040.

| **Year** | **China** | | **India** | | **the United States** | |
| --- | --- | --- | --- | --- | --- | --- |
|  | **Number** | **ASR** | **Number** | **ASR** | **Number** | **ASR** |
| 1994 | 39112 | 4.295201 | 3375 | 0.665363 | 8246 | 3.840915 |
| 1995 | 38868 | 4.205695 | 3509 | 0.674123 | 9125 | 4.197459 |
| 1996 | 38412 | 4.103317 | 3747 | 0.703494 | 9554 | 4.346341 |
| 1997 | 36956 | 3.901556 | 4021 | 0.737953 | 10006 | 4.517993 |
| 1998 | 35473 | 3.702133 | 4280 | 0.764237 | 11025 | 4.936559 |
| 1999 | 32874 | 3.392498 | 4490 | 0.779267 | 12568 | 5.577903 |
| 2000 | 30254 | 3.082152 | 4690 | 0.790857 | 14198 | 6.253503 |
| 2001 | 26714 | 2.685147 | 4895 | 0.804188 | 16072 | 7.012935 |
| 2002 | 22976 | 2.276194 | 5093 | 0.818606 | 18439 | 7.97985 |
| 2003 | 17746 | 1.730233 | 5072 | 0.794724 | 20692 | 8.892743 |
| 2004 | 14608 | 1.404074 | 5059 | 0.772869 | 22564 | 9.618877 |
| 2005 | 13108 | 1.240619 | 5502 | 0.818508 | 24982 | 10.561848 |
| 2006 | 12025 | 1.124656 | 5050 | 0.734838 | 27271 | 11.449979 |
| 2007 | 11317 | 1.046935 | 4997 | 0.709753 | 28819 | 12.015768 |
| 2008 | 10670 | 0.975179 | 5064 | 0.702114 | 30357 | 12.557124 |
| 2009 | 10038 | 0.905779 | 4747 | 0.64191 | 31760 | 13.038281 |
| 2010 | 9378 | 0.836268 | 4585 | 0.605731 | 33368 | 13.597458 |
| 2011 | 8835 | 0.779996 | 4446 | 0.573554 | 35481 | 14.355242 |
| 2012 | 8349 | 0.729875 | 4457 | 0.562183 | 37395 | 15.020027 |
| 2013 | 7793 | 0.673206 | 4235 | 0.522749 | 40022 | 15.967497 |
| 2014 | 7397 | 0.630534 | 4164 | 0.502378 | 43391 | 17.224489 |
| 2015 | 7133 | 0.600114 | 4151 | 0.491643 | 48274 | 19.120028 |
| 2016 | 7019 | 0.582978 | 4318 | 0.499568 | 54469 | 21.568938 |
| 2017 | 6967 | 0.574029 | 4352 | 0.49397 | 59771 | 23.535366 |
| 2018 | 6934 | 0.570154 | 4470 | 0.495484 | 62895 | 24.53323 |
| 2019 | 6779 | 0.558436 | 4758 | 0.514053 | 68505 | 26.567985 |
| 2020 | 6766 | 0.557411 | 5083 | 0.537232 | 80377 | 31.25503 |
| 2021 | 7349 | 0.599253 | 5874 | 0.602568 | 90405 | 35.055727 |
| 2022 | 8313 | 0.670968 | 5878 | 0.595514 | 96007 | 36.902735 |
| 2023 | 8186 | 0.661417 | 6238 | 0.617646 | 95333 | 36.41835 |
| 2024 | 7864 | 0.613644 | 6511 | 0.631838 | 111912 | 42.626433 |
| 2025 | 7933 | 0.616399 | 6842 | 0.650719 | 120924 | 45.786555 |
| 2026 | 8002 | 0.619196 | 7181 | 0.669597 | 130029 | 48.948751 |
| 2027 | 8063 | 0.621858 | 7493 | 0.685183 | 139075 | 51.999802 |
| 2028 | 8129 | 0.624537 | 7814 | 0.700788 | 148186 | 55.052439 |
| 2029 | 8201 | 0.627292 | 8144 | 0.716418 | 157358 | 58.107145 |
| 2030 | 8275 | 0.630103 | 8482 | 0.732057 | 166607 | 61.163947 |
| 2031 | 8352 | 0.63294 | 8825 | 0.74769 | 175942 | 64.222702 |
| 2032 | 8411 | 0.634641 | 9115 | 0.758754 | 183646 | 66.612247 |
| 2033 | 8461 | 0.636312 | 9410 | 0.769817 | 191409 | 69.002932 |
| 2034 | 8507 | 0.638002 | 9712 | 0.780882 | 199232 | 71.395249 |
| 2035 | 8551 | 0.63972 | 10019 | 0.791941 | 207122 | 73.789193 |
| 2036 | 8596 | 0.641453 | 10328 | 0.802997 | 215079 | 76.184584 |
| 2037 | 8619 | 0.641597 | 10559 | 0.808201 | 219933 | 77.466256 |
| 2038 | 8638 | 0.641712 | 10793 | 0.813432 | 224794 | 78.748242 |
| 2039 | 8655 | 0.641829 | 11032 | 0.818685 | 229665 | 80.03109 |
| 2040 | 8673 | 0.641956 | 11273 | 0.823943 | 234558 | 81.314703 |

Supplementary Table S9**.** Projected case number and age-standardized rate (ASR) of disability-adjusted life years (DALYs) for drug use disorders to 2040.

| **Year** | **China** | | **India** | | **the United States** | |
| --- | --- | --- | --- | --- | --- | --- |
|  | **Number** | **ASR** | **Number** | **ASR** | **Number** | **ASR** |
| 1994 | 4146668 | 368.407086 | 581730 | 85.221486 | 1105853 | 482.6147 |
| 1995 | 4128087 | 363.975312 | 598600 | 85.732002 | 1159987 | 501.891732 |
| 1996 | 4074184 | 357.678849 | 624756 | 87.515319 | 1192475 | 512.012648 |
| 1997 | 3952395 | 346.063952 | 659508 | 90.256815 | 1230540 | 525.097289 |
| 1998 | 3825315 | 334.199213 | 697766 | 93.169353 | 1300241 | 550.6636 |
| 1999 | 3639557 | 317.200925 | 732585 | 95.35482 | 1399584 | 587.870421 |
| 2000 | 3465035 | 301.043341 | 760701 | 96.547135 | 1506797 | 628.657182 |
| 2001 | 3241057 | 280.75399 | 778487 | 96.487288 | 1632954 | 674.643426 |
| 2002 | 2995333 | 258.623376 | 787959 | 95.49948 | 1784917 | 731.161881 |
| 2003 | 2672919 | 229.887794 | 782507 | 92.703458 | 1932035 | 786.209694 |
| 2004 | 2456840 | 210.015218 | 776252 | 89.902288 | 2063179 | 833.687229 |
| 2005 | 2325851 | 196.929242 | 793691 | 89.9119 | 2221738 | 891.41953 |
| 2006 | 2195695 | 184.00415 | 765067 | 84.818423 | 2381977 | 949.119817 |
| 2007 | 2050836 | 170.011487 | 752221 | 81.722425 | 2511964 | 994.718805 |
| 2008 | 1903424 | 156.066117 | 744946 | 79.34722 | 2647538 | 1041.31053 |
| 2009 | 1776643 | 144.152346 | 725612 | 75.679527 | 2783672 | 1087.639886 |
| 2010 | 1696386 | 136.4409 | 721193 | 73.660199 | 2936249 | 1140.433872 |
| 2011 | 1654745 | 132.372508 | 736633 | 73.576909 | 3138083 | 1211.16293 |
| 2012 | 1615663 | 128.922391 | 775531 | 75.689375 | 3361879 | 1289.724495 |
| 2013 | 1572436 | 125.508218 | 812355 | 77.446817 | 3641322 | 1389.176304 |
| 2014 | 1534297 | 122.899587 | 853401 | 79.569243 | 3974538 | 1508.764038 |
| 2015 | 1504018 | 121.247701 | 882430 | 80.634084 | 4389392 | 1660.256307 |
| 2016 | 1483034 | 120.65032 | 911274 | 81.705069 | 4910062 | 1852.389971 |
| 2017 | 1471346 | 121.238923 | 938415 | 82.590062 | 5411809 | 2034.323828 |
| 2018 | 1465888 | 122.725932 | 974781 | 84.190569 | 5794537 | 2170.18178 |
| 2019 | 1455697 | 124.07518 | 1018605 | 86.398471 | 6269395 | 2342.780563 |
| 2020 | 1447451 | 125.501277 | 1055974 | 88.087544 | 6985587 | 2611.323103 |
| 2021 | 1483686 | 129.931974 | 1131218 | 92.915442 | 7540760 | 2814.346176 |
| 2022 | 1532342 | 135.106573 | 1165244 | 94.262112 | 7832364 | 2907.110887 |
| 2023 | 1502237 | 133.997049 | 1184024 | 94.394209 | 7796439 | 2879.855965 |
| 2024 | 1566796 | 135.634833 | 1237419 | 96.772837 | 9642291 | 3500.167058 |
| 2025 | 1569410 | 137.732815 | 1277311 | 98.587474 | 10251533 | 3698.305159 |
| 2026 | 1572516 | 139.834394 | 1317577 | 100.400309 | 10802629 | 3868.317066 |
| 2027 | 1579517 | 142.353776 | 1350319 | 101.635119 | 11356795 | 4038.369743 |
| 2028 | 1587482 | 144.870841 | 1383335 | 102.867976 | 11914198 | 4208.47648 |
| 2029 | 1595683 | 147.388686 | 1416625 | 104.099182 | 12475674 | 4378.639402 |
| 2030 | 1604837 | 149.906518 | 1449911 | 105.329274 | 13041721 | 4548.854406 |
| 2031 | 1615912 | 152.422774 | 1482987 | 106.558476 | 13475548 | 4667.722638 |
| 2032 | 1627079 | 154.57625 | 1509202 | 107.354907 | 13911034 | 4786.608045 |
| 2033 | 1636404 | 156.724597 | 1535383 | 108.150433 | 14348894 | 4905.517645 |
| 2034 | 1645556 | 158.868539 | 1561579 | 108.94508 | 14790465 | 5024.4558 |
| 2035 | 1655682 | 161.009824 | 1587361 | 109.739434 | 15236114 | 5143.421371 |
| 2036 | 1668096 | 163.149697 | 1612360 | 110.533869 | 15496171 | 5198.853221 |
| 2037 | 1678660 | 164.491063 | 1629260 | 110.897585 | 15756468 | 5254.27877 |
| 2038 | 1682766 | 165.83133 | 1645737 | 111.261838 | 16017375 | 5309.701743 |
| 2039 | 1684874 | 167.168749 | 1661913 | 111.62648 | 9038317 | 3302.105031 |
| 2040 | 1685135 | 168.50458 | 1677578 | 111.991334 | 16280266 | 5365.123872 |

Supplementary Table S10. Sensitivity analysis of the Nordpred age-period-cohort model: Predictions of drug use disorders in China, India, and the United States using original data, lower bound, and upper bound of case numbers.

| **Year** | **Case numbers of incidence** | | | **Case numbers of prevalence** | | | **Case numbers of deaths** | | | **Case numbers of DALYs** | | |
| --- | --- | --- | --- | --- | --- | --- | --- | --- | --- | --- | --- | --- |
|  | Original Prediction | Lower Bound Prediction | Upper Bound Prediction | Original Prediction | Lower Bound Prediction | Upper Bound Prediction | Original Prediction | Lower Bound Prediction | Upper Bound Prediction | Original Prediction | Lower Bound Prediction | Upper Bound Prediction |
| **China** | | | | | | | | | | | | |
| 1994 | 3290859 | 2440090 | 4311751 | 12115087 | 9649080 | 15455431 | 39112 | 23982 | 62300 | 4146668 | 2961302 | 5591419 |
| 1995 | 3302155 | 2443617 | 4337699 | 12100998 | 9653743 | 15417708 | 38868 | 23576 | 60949 | 4128087 | 2965061 | 5550347 |
| 1996 | 3303193 | 2440601 | 4342563 | 11977961 | 9543642 | 15275353 | 38412 | 23323 | 59695 | 4074184 | 2923646 | 5410005 |
| 1997 | 3300886 | 2434220 | 4347862 | 11782516 | 9375601 | 15042229 | 36956 | 22587 | 56500 | 3952395 | 2849705 | 5217082 |
| 1998 | 3301881 | 2428788 | 4361972 | 11556217 | 9175558 | 14761538 | 35473 | 22066 | 54128 | 3825315 | 2762231 | 5078907 |
| 1999 | 3306088 | 2426553 | 4377549 | 11328237 | 8981453 | 14484249 | 32874 | 20788 | 49453 | 3639557 | 2642517 | 4828359 |
| 2000 | 3319552 | 2431226 | 4404237 | 11122789 | 8800334 | 14242814 | 30254 | 19371 | 45614 | 3465035 | 2533729 | 4586476 |
| 2001 | 3332349 | 2439282 | 4426075 | 10879873 | 8590649 | 13948907 | 26714 | 17117 | 40274 | 3241057 | 2385067 | 4287802 |
| 2002 | 3340643 | 2444357 | 4436657 | 10566985 | 8326055 | 13581975 | 22976 | 14865 | 34378 | 2995333 | 2205719 | 3952636 |
| 2003 | 3346950 | 2449381 | 4443698 | 10237415 | 8046423 | 13182881 | 17746 | 11492 | 26683 | 2672919 | 1946893 | 3525638 |
| 2004 | 3349857 | 2449032 | 4445568 | 9933883 | 7779711 | 12821896 | 14608 | 9540 | 21877 | 2456840 | 1787369 | 3246934 |
| 2005 | 3352402 | 2446259 | 4451005 | 9686503 | 7555838 | 12537520 | 13108 | 8600 | 19341 | 2325851 | 1678306 | 3066061 |
| 2006 | 3329144 | 2424685 | 4417269 | 9390079 | 7297558 | 12177648 | 12025 | 7862 | 17758 | 2195695 | 1567069 | 2914098 |
| 2007 | 3271052 | 2376066 | 4343296 | 8985594 | 6940441 | 11694019 | 11317 | 7429 | 16579 | 2050836 | 1461413 | 2700580 |
| 2008 | 3196799 | 2314393 | 4251783 | 8564047 | 6576289 | 11168054 | 10670 | 7065 | 15556 | 1903424 | 1353848 | 2508058 |
| 2009 | 3125311 | 2255885 | 4168897 | 8222807 | 6288104 | 10743584 | 10038 | 6644 | 14672 | 1776643 | 1260240 | 2352502 |
| 2010 | 3077873 | 2219739 | 4119698 | 8065172 | 6148305 | 10554369 | 9378 | 6231 | 13580 | 1696386 | 1203006 | 2251418 |
| 2011 | 3042189 | 2192170 | 4075033 | 8021940 | 6117280 | 10475960 | 8835 | 5932 | 12842 | 1654745 | 1173092 | 2194868 |
| 2012 | 2998351 | 2156944 | 4016207 | 7960595 | 6075508 | 10377312 | 8349 | 5661 | 11988 | 1615663 | 1148642 | 2145906 |
| 2013 | 2950585 | 2119466 | 3956367 | 7879858 | 6019099 | 10256479 | 7793 | 5403 | 11024 | 1572436 | 1109745 | 2100009 |
| 2014 | 2903146 | 2082051 | 3897472 | 7780154 | 5947151 | 10118054 | 7397 | 5160 | 10505 | 1534297 | 1077805 | 2038138 |
| 2015 | 2859792 | 2048405 | 3845767 | 7668099 | 5867362 | 9970970 | 7133 | 4980 | 10069 | 1504018 | 1058628 | 2008176 |
| 2016 | 2824766 | 2023811 | 3803407 | 7599487 | 5832176 | 9866295 | 7019 | 4878 | 9837 | 1483034 | 1046389 | 1977747 |
| 2017 | 2797524 | 2003534 | 3769649 | 7593135 | 5840125 | 9852133 | 6967 | 4794 | 9955 | 1471346 | 1036680 | 1956835 |
| 2018 | 2774993 | 1986553 | 3742796 | 7601534 | 5838219 | 9862874 | 6934 | 4750 | 10065 | 1465888 | 1033913 | 1958383 |
| 2019 | 2755134 | 1972842 | 3723028 | 7584491 | 5831190 | 9839397 | 6779 | 4630 | 9853 | 1455697 | 1027384 | 1952434 |
| 2020 | 2735449 | 1958813 | 3700145 | 7512648 | 5782431 | 9745518 | 6766 | 4554 | 9924 | 1447451 | 1022357 | 1941634 |
| 2021 | 2738175 | 1960564 | 3708468 | 7501142 | 5754944 | 9720539 | 7349 | 4704 | 11057 | 1483686 | 1043693 | 1988101 |
| 2022 | 2743613 | 1958702 | 3728986 | 7488195 | 5731210 | 9720826 | 8313 | 4901 | 13320 | 1532342 | 1074141 | 2062962 |
| 2023 | 2720246 | 1937224 | 3685425 | 7348874 | 5621718 | 9545126 | 8186 | 4579 | 13474 | 1502237 | 1049732 | 2026727 |
| 2024 | 2836594 | 2020103 | 3848353 | 7899905 | 6047305 | 10245354 | 7864 | 4623 | 12712 | 1566796 | 1096878 | 2117690 |
| 2025 | 2827524 | 2009592 | 3839598 | 7911321 | 6047927 | 10262043 | 7933 | 4568 | 13019 | 1569410 | 1097294 | 2125235 |
| 2026 | 2818560 | 1999353 | 3830652 | 7929326 | 6053072 | 10287670 | 8002 | 4513 | 13328 | 1572516 | 1097994 | 2133381 |
| 2027 | 2810535 | 1989714 | 3821658 | 7959975 | 6065615 | 10327467 | 8063 | 4481 | 13582 | 1579517 | 1101313 | 2145966 |
| 2028 | 2799625 | 1979114 | 3806902 | 7996681 | 6083605 | 10373616 | 8129 | 4452 | 13847 | 1587482 | 1105370 | 2159597 |
| 2029 | 2786959 | 1967790 | 3788798 | 8037270 | 6104533 | 10424243 | 8201 | 4425 | 14123 | 1595683 | 1109622 | 2173372 |
| 2030 | 2774577 | 1957267 | 3770204 | 8085986 | 6131236 | 10485612 | 8275 | 4399 | 14407 | 1604837 | 1114496 | 2188338 |
| 2031 | 2764663 | 1949162 | 3754104 | 8148112 | 6167479 | 10565216 | 8352 | 4373 | 14695 | 1615912 | 1120608 | 2205872 |
| 2032 | 2759557 | 1945344 | 3743867 | 8208337 | 6203423 | 10642882 | 8411 | 4364 | 14901 | 1627079 | 1127151 | 2222772 |
| 2033 | 2746470 | 1935761 | 3724028 | 8254172 | 6230533 | 10700268 | 8461 | 4349 | 15087 | 1636404 | 1132321 | 2237250 |
| 2034 | 2730082 | 1924111 | 3699862 | 8299171 | 6257493 | 10756381 | 8507 | 4333 | 15266 | 1645556 | 1137254 | 2251538 |
| 2035 | 2712499 | 1911769 | 3674143 | 8350130 | 6289534 | 10819595 | 8551 | 4317 | 15440 | 1655682 | 1142708 | 2267209 |
| 2036 | 2695787 | 1900046 | 3649604 | 8414387 | 6332422 | 10898842 | 8596 | 4300 | 15614 | 1668096 | 1149561 | 2286057 |
| 2037 | 2682325 | 1890422 | 3629321 | 8475636 | 6375008 | 10974938 | 8619 | 4297 | 15693 | 1678660 | 1155434 | 2301648 |
| 2038 | 2665039 | 1877743 | 3604749 | 8495793 | 6387122 | 10997697 | 8638 | 4291 | 15762 | 1682766 | 1157264 | 2308303 |
| 2039 | 2645848 | 1863628 | 3577985 | 8502975 | 6389926 | 11003126 | 8655 | 4286 | 15829 | 1684874 | 1157815 | 2312187 |
| 2040 | 2625248 | 1848364 | 3549873 | 8497111 | 6383652 | 10990756 | 8673 | 4280 | 15896 | 1685135 | 1157231 | 2313545 |
| **India** | | | | | | | | | | | | |
| 1994 | 1050464 | 696375 | 1525445 | 3416138 | 2331269 | 4961203 | 3375 | 1929 | 5611 | 581730 | 408969 | 790145 |
| 1995 | 1075283 | 712664 | 1563328 | 3494754 | 2376680 | 5084634 | 3509 | 2010 | 5706 | 598600 | 421012 | 806793 |
| 1996 | 1100553 | 732919 | 1594891 | 3578618 | 2437761 | 5187867 | 3747 | 2180 | 6082 | 624756 | 438165 | 838655 |
| 1997 | 1127578 | 753364 | 1626371 | 3672292 | 2513779 | 5290855 | 4021 | 2331 | 6480 | 659508 | 460939 | 888570 |
| 1998 | 1155900 | 777292 | 1654938 | 3771608 | 2599012 | 5400129 | 4280 | 2437 | 6991 | 697766 | 489616 | 931023 |
| 1999 | 1185025 | 799064 | 1689695 | 3872497 | 2678778 | 5523429 | 4490 | 2553 | 7368 | 732585 | 508558 | 979679 |
| 2000 | 1214496 | 819251 | 1729402 | 3970904 | 2731288 | 5653951 | 4690 | 2672 | 7802 | 760701 | 527523 | 1018098 |
| 2001 | 1241409 | 837171 | 1767626 | 4055208 | 2787424 | 5776789 | 4895 | 2821 | 8063 | 778487 | 541270 | 1049069 |
| 2002 | 1265296 | 852375 | 1802708 | 4122935 | 2828705 | 5882273 | 5093 | 2957 | 8250 | 787959 | 547735 | 1060896 |
| 2003 | 1287233 | 865777 | 1835790 | 4179908 | 2860617 | 5976747 | 5072 | 2996 | 8078 | 782507 | 546031 | 1053288 |
| 2004 | 1308422 | 878351 | 1868227 | 4232975 | 2889463 | 6067359 | 5059 | 3037 | 8047 | 776252 | 544548 | 1044968 |
| 2005 | 1330291 | 891774 | 1901367 | 4289786 | 2922065 | 6163201 | 5502 | 3350 | 8765 | 793691 | 563302 | 1060668 |
| 2006 | 1352810 | 906144 | 1936122 | 4357660 | 2970657 | 6278344 | 5050 | 3123 | 7943 | 765067 | 541377 | 1021686 |
| 2007 | 1376275 | 919281 | 1973251 | 4438096 | 3013220 | 6429561 | 4997 | 3075 | 7854 | 752221 | 531111 | 1011994 |
| 2008 | 1401168 | 932789 | 2015550 | 4527204 | 3059288 | 6571283 | 5064 | 3137 | 7946 | 744946 | 539208 | 994485 |
| 2009 | 1427948 | 947648 | 2062692 | 4620861 | 3108600 | 6748000 | 4747 | 2908 | 7451 | 725612 | 522446 | 978738 |
| 2010 | 1457071 | 965663 | 2108997 | 4714516 | 3161486 | 6904090 | 4585 | 2840 | 7149 | 721193 | 520126 | 964651 |
| 2011 | 1494657 | 992981 | 2159321 | 4833831 | 3251307 | 7060729 | 4446 | 2772 | 6853 | 736633 | 530982 | 978501 |
| 2012 | 1543498 | 1029813 | 2222789 | 4994927 | 3381149 | 7258123 | 4457 | 2793 | 6794 | 775531 | 557968 | 1033805 |
| 2013 | 1597658 | 1070495 | 2292116 | 5174452 | 3527125 | 7473260 | 4235 | 2707 | 6315 | 812355 | 584901 | 1078317 |
| 2014 | 1650936 | 1110463 | 2361350 | 5347206 | 3666925 | 7682068 | 4164 | 2688 | 6173 | 853401 | 611628 | 1138534 |
| 2015 | 1696756 | 1143679 | 2421014 | 5486115 | 3775044 | 7859972 | 4151 | 2690 | 6115 | 882430 | 630735 | 1172764 |
| 2016 | 1735126 | 1178309 | 2462768 | 5578860 | 3871769 | 7971180 | 4318 | 2796 | 6377 | 911274 | 650856 | 1215852 |
| 2017 | 1771764 | 1210335 | 2498166 | 5644986 | 3958779 | 8033304 | 4352 | 2778 | 6487 | 938415 | 671254 | 1244374 |
| 2018 | 1807954 | 1243848 | 2527900 | 5702035 | 4035879 | 8095125 | 4470 | 2836 | 6752 | 974781 | 698326 | 1290912 |
| 2019 | 1845016 | 1274744 | 2572681 | 5768196 | 4109166 | 8162644 | 4758 | 3023 | 7232 | 1018605 | 734218 | 1347711 |
| 2020 | 1883746 | 1299578 | 2627317 | 5860525 | 4176611 | 8288360 | 5083 | 3204 | 7778 | 1055974 | 750848 | 1402016 |
| 2021 | 1932981 | 1335370 | 2689810 | 6012494 | 4295898 | 8477671 | 5874 | 3571 | 9176 | 1131218 | 805715 | 1498456 |
| 2022 | 1977794 | 1368359 | 2748653 | 6150912 | 4405170 | 8650872 | 5878 | 3343 | 9590 | 1165244 | 823305 | 1550984 |
| 2023 | 1981112 | 1369858 | 2753163 | 6073948 | 4350412 | 8499911 | 6238 | 3407 | 10539 | 1184024 | 835512 | 1586408 |
| 2024 | 2027290 | 1416918 | 2808666 | 6182239 | 4498447 | 8676283 | 6511 | 3699 | 10806 | 1237419 | 879058 | 1646523 |
| 2025 | 2054758 | 1441531 | 2841593 | 6226693 | 4557834 | 8727410 | 6842 | 3835 | 11490 | 1277311 | 907160 | 1700095 |
| 2026 | 2081742 | 1465988 | 2873348 | 6268725 | 4615716 | 8774634 | 7181 | 3974 | 12192 | 1317577 | 935507 | 1754180 |
| 2027 | 2104861 | 1487570 | 2900664 | 6304808 | 4663421 | 8822750 | 7493 | 4100 | 12847 | 1350319 | 959468 | 1799377 |
| 2028 | 2127690 | 1509075 | 2927242 | 6338757 | 4710018 | 8867536 | 7814 | 4230 | 13520 | 1383335 | 983594 | 1844955 |
| 2029 | 2150141 | 1530446 | 2953003 | 6370606 | 4755295 | 8909254 | 8144 | 4362 | 14216 | 1416625 | 1007887 | 1890940 |
| 2030 | 2171628 | 1551147 | 2977266 | 6398935 | 4798173 | 8945516 | 8482 | 4498 | 14927 | 1449911 | 1032145 | 1936953 |
| 2031 | 2191715 | 1570716 | 2999564 | 6422636 | 4837890 | 8974283 | 8825 | 4634 | 15653 | 1482987 | 1056214 | 1982693 |
| 2032 | 2206510 | 1585197 | 3016707 | 6443289 | 4870179 | 8997790 | 9115 | 4752 | 16259 | 1509202 | 1075607 | 2019362 |
| 2033 | 2220423 | 1598873 | 3032704 | 6461055 | 4900690 | 9016377 | 9410 | 4871 | 16877 | 1535383 | 1094949 | 2055962 |
| 2034 | 2233619 | 1611983 | 3047680 | 6476515 | 4929597 | 9031365 | 9712 | 4993 | 17510 | 1561579 | 1114278 | 2092594 |
| 2035 | 2245737 | 1624319 | 3061075 | 6487990 | 4955279 | 9040873 | 10019 | 5116 | 18153 | 1587361 | 1133275 | 2128701 |
| 2036 | 2256539 | 1635732 | 3072533 | 6494074 | 4976393 | 9043339 | 10328 | 5240 | 18804 | 1612360 | 1151667 | 2163794 |
| 2037 | 2259759 | 1641323 | 3076444 | 6492926 | 4986673 | 9036863 | 10559 | 5338 | 19265 | 1629260 | 1164109 | 2187651 |
| 2038 | 2261733 | 1646063 | 3078462 | 6487957 | 4994082 | 9024746 | 10793 | 5436 | 19732 | 1645737 | 1176223 | 2210931 |
| 2039 | 2262492 | 1649976 | 3078663 | 6479854 | 4999125 | 9007876 | 11032 | 5537 | 20209 | 1661913 | 1188102 | 2233818 |
| 2040 | 2261948 | 1652997 | 3076948 | 6468249 | 5001423 | 8985929 | 11273 | 5638 | 20691 | 1677578 | 1199580 | 2256040 |
| **the United States** | | | | | | | | | | | | |
| 1994 | 1055050 | 743796 | 1471368 | 5113781 | 4028768 | 6481666 | 8246 | 6067 | 11260 | 1105853 | 835719 | 1375989 |
| 1995 | 1069750 | 755035 | 1491907 | 5137788 | 4054517 | 6513513 | 9125 | 6742 | 12263 | 1159987 | 876405 | 1441122 |
| 1996 | 1083799 | 767681 | 1511444 | 5167852 | 4093168 | 6542852 | 9554 | 7098 | 12977 | 1192475 | 906672 | 1477879 |
| 1997 | 1094917 | 778102 | 1525527 | 5197782 | 4131115 | 6573717 | 10006 | 7449 | 13451 | 1230540 | 939774 | 1524685 |
| 1998 | 1105267 | 785834 | 1536657 | 5237428 | 4176978 | 6614293 | 11025 | 8195 | 14689 | 1300241 | 996028 | 1609187 |
| 1999 | 1116941 | 794064 | 1552971 | 5294325 | 4235145 | 6673953 | 12568 | 9367 | 16700 | 1399584 | 1082868 | 1727586 |
| 2000 | 1129978 | 802789 | 1570874 | 5372850 | 4312282 | 6760135 | 14198 | 10569 | 18678 | 1506797 | 1173075 | 1863456 |
| 2001 | 1149094 | 818095 | 1593498 | 5501523 | 4432671 | 6906051 | 16072 | 11933 | 21150 | 1632954 | 1280604 | 2024096 |
| 2002 | 1166798 | 832117 | 1615011 | 5634661 | 4558115 | 7055984 | 18439 | 13645 | 24145 | 1784917 | 1407870 | 2204722 |
| 2003 | 1182986 | 845550 | 1634118 | 5768443 | 4682425 | 7206318 | 20692 | 15225 | 26928 | 1932035 | 1517594 | 2392546 |
| 2004 | 1201039 | 860306 | 1656176 | 5916928 | 4820006 | 7369831 | 22564 | 16473 | 29416 | 2063179 | 1612067 | 2560199 |
| 2005 | 1218274 | 875123 | 1677499 | 6066494 | 4958732 | 7535014 | 24982 | 18183 | 32596 | 2221738 | 1731695 | 2758136 |
| 2006 | 1238661 | 892221 | 1700747 | 6240224 | 5108073 | 7736420 | 27271 | 19609 | 35742 | 2381977 | 1851491 | 2946142 |
| 2007 | 1262123 | 914078 | 1728060 | 6441208 | 5277670 | 7975813 | 28819 | 20874 | 37676 | 2511964 | 1946073 | 3103216 |
| 2008 | 1286925 | 937814 | 1757549 | 6663015 | 5462269 | 8242068 | 30357 | 22457 | 39447 | 2647538 | 2042300 | 3262614 |
| 2009 | 1310662 | 959101 | 1786322 | 6894101 | 5657799 | 8517084 | 31760 | 23327 | 41185 | 2783672 | 2171281 | 3413097 |
| 2010 | 1331004 | 976542 | 1809577 | 7121482 | 5854715 | 8772600 | 33368 | 24486 | 43297 | 2936249 | 2309721 | 3587016 |
| 2011 | 1355195 | 997652 | 1836246 | 7394246 | 6099307 | 9062713 | 35481 | 26098 | 46039 | 3138083 | 2492828 | 3826329 |
| 2012 | 1387579 | 1026078 | 1873479 | 7741100 | 6407703 | 9438628 | 37395 | 27898 | 48689 | 3361879 | 2676829 | 4085736 |
| 2013 | 1424539 | 1057233 | 1916837 | 8140059 | 6761498 | 9881277 | 40022 | 30251 | 52113 | 3641322 | 2906593 | 4425665 |
| 2014 | 1463065 | 1088396 | 1962424 | 8575634 | 7136690 | 10384679 | 43391 | 33112 | 55858 | 3974538 | 3181070 | 4810967 |
| 2015 | 1498794 | 1118029 | 2005922 | 9019905 | 7523281 | 10901928 | 48274 | 37063 | 62091 | 4389392 | 3530640 | 5338341 |
| 2016 | 1537697 | 1152307 | 2049099 | 9585800 | 8047455 | 11509329 | 54469 | 41743 | 69782 | 4910062 | 3967115 | 5952087 |
| 2017 | 1583285 | 1192178 | 2100049 | 10308643 | 8714209 | 12288612 | 59771 | 45755 | 76628 | 5411809 | 4374909 | 6547268 |
| 2018 | 1629089 | 1233332 | 2152255 | 11061261 | 9408396 | 13093990 | 62895 | 48089 | 80300 | 5794537 | 4683984 | 7008674 |
| 2019 | 1669748 | 1268735 | 2195603 | 11725754 | 10012486 | 13821565 | 68505 | 51853 | 87771 | 6269395 | 5079835 | 7587194 |
| 2020 | 1699420 | 1292607 | 2225498 | 12173182 | 10412293 | 14315456 | 80377 | 60758 | 104137 | 6985587 | 5634534 | 8490122 |
| 2021 | 1731083 | 1318081 | 2262195 | 12496729 | 10683837 | 14687960 | 90405 | 67821 | 117982 | 7540760 | 6048055 | 9200799 |
| 2022 | 1759299 | 1340440 | 2303528 | 12723615 | 10869927 | 14964117 | 96007 | 72245 | 124534 | 7832364 | 6287786 | 9532712 |
| 2023 | 1766916 | 1340693 | 2321914 | 12796839 | 10913443 | 15077472 | 95333 | 72322 | 123014 | 7796439 | 6281091 | 9462133 |
| 2024 | 1824561 | 1394050 | 2377614 | 14026394 | 12072177 | 16368496 | 111912 | 83704 | 145686 | 9642291 | 7270772 | 11003729 |
| 2025 | 1856060 | 1419508 | 2414871 | 14567817 | 12566182 | 16958070 | 120924 | 90253 | 157622 | 10251533 | 7755201 | 11744048 |
| 2026 | 1887841 | 1445117 | 2452560 | 15111550 | 13062385 | 17550080 | 130029 | 96871 | 169677 | 10802629 | 8243949 | 12490826 |
| 2027 | 1918598 | 1469337 | 2489454 | 15573560 | 13477401 | 18057780 | 139075 | 103390 | 181709 | 11356795 | 8686393 | 13166373 |
| 2028 | 1949197 | 1493229 | 2526452 | 16034426 | 13891429 | 18563641 | 148186 | 109954 | 193828 | 11914198 | 9131278 | 13845739 |
| 2029 | 1979490 | 1516731 | 2563270 | 16493520 | 14304181 | 19066767 | 157358 | 116563 | 206027 | 12475674 | 9578845 | 14529066 |
| 2030 | 2009903 | 1540287 | 2600306 | 16953648 | 14717831 | 19570971 | 166607 | 123226 | 218326 | 13041721 | 10029783 | 15217408 |
| 2031 | 2040834 | 1564316 | 2637929 | 17417665 | 15134522 | 20080176 | 175942 | 129951 | 230739 | 13475548 | 10484459 | 15911435 |
| 2032 | 2066151 | 1583907 | 2668597 | 17763754 | 15442503 | 20460600 | 183646 | 135488 | 240991 | 13911034 | 10833013 | 16443363 |
| 2033 | 2091818 | 1603935 | 2699527 | 18111546 | 15751174 | 20844250 | 191409 | 141067 | 251325 | 14348894 | 11182869 | 16977517 |
| 2034 | 2117625 | 1624218 | 2730478 | 18460341 | 16060217 | 21229898 | 199232 | 146686 | 261739 | 14790465 | 11534688 | 17514717 |
| 2035 | 2143586 | 1644740 | 2761474 | 18811971 | 16371589 | 21619111 | 207122 | 152353 | 272245 | 15236114 | 11889564 | 18056574 |
| 2036 | 2169572 | 1665355 | 2792372 | 19167300 | 16686375 | 22012296 | 215079 | 158067 | 282842 | 15496171 | 12247755 | 18603552 |
| 2037 | 2185991 | 1678269 | 2812154 | 19376980 | 16871801 | 22242510 | 219933 | 161577 | 289269 | 15756468 | 12457459 | 18920418 |
| 2038 | 2202093 | 1690924 | 2831500 | 19587586 | 17058167 | 22473290 | 224794 | 165089 | 295711 | 16017375 | 12667231 | 19237815 |
| 2039 | 2217749 | 1703260 | 2850201 | 19797927 | 17244499 | 22703318 | 229665 | 168605 | 302170 | 9038317 | 12877450 | 19556165 |
| 2040 | 2233057 | 1715376 | 2868363 | 20008911 | 17431777 | 22933511 | 234558 | 172135 | 308662 | 16280266 | 13089294 | 19877048 |

Supplementary Table S11**.** Sensitivity analysis of the Nordpred age-period-cohort model: Predictions of drug use disorders in China, India, and the United States using original data, lower bound, and upper bound of age-standardized rate (ASR).

| **Year** | **ASIR** | | | **ASPR** | | | **ASMR** | | | **ASDR** | | |
| --- | --- | --- | --- | --- | --- | --- | --- | --- | --- | --- | --- | --- |
|  | Original Prediction | Lower Bound Prediction | Upper Bound Prediction | Original Prediction | Lower Bound Prediction | Upper Bound Prediction | Original Prediction | Lower Bound Prediction | Upper Bound Prediction | Original Prediction | Lower Bound Prediction | Upper Bound Prediction |
| **China** | | | | | | | | | | | | |
| 1994 | 294.086749 | 217.13081 | 387.014809 | 1027.273822 | 817.15612 | 1310.544907 | 4.295201 | 2.634611 | 6.81929 | 368.407086 | 262.879753 | 497.756432 |
| 1995 | 293.708587 | 216.682945 | 387.190961 | 1025.983103 | 816.951555 | 1308.025295 | 4.205695 | 2.553197 | 6.594882 | 363.975312 | 261.008236 | 490.609789 |
| 1996 | 293.028218 | 216.09387 | 386.191542 | 1019.916183 | 810.766849 | 1301.97622 | 4.103317 | 2.496871 | 6.379819 | 357.678849 | 256.400434 | 476.21079 |
| 1997 | 292.109121 | 215.299379 | 385.232734 | 1010.099489 | 801.492818 | 1291.283043 | 3.901556 | 2.390473 | 5.968912 | 346.063952 | 249.153153 | 458.051966 |
| 1998 | 290.992022 | 214.197394 | 384.385968 | 997.791643 | 789.645817 | 1276.69157 | 3.702133 | 2.300805 | 5.654903 | 334.199213 | 241.027657 | 444.525447 |
| 1999 | 289.726928 | 213.018519 | 383.106645 | 984.233197 | 777.674945 | 1260.837907 | 3.392498 | 2.138258 | 5.124914 | 317.200925 | 229.896284 | 421.505971 |
| 2000 | 288.36539 | 211.707972 | 381.667451 | 970.746839 | 765.469797 | 1245.355593 | 3.082152 | 1.963633 | 4.661621 | 301.043341 | 219.728282 | 399.191583 |
| 2001 | 286.429911 | 210.247507 | 379.234036 | 952.792401 | 749.80953 | 1223.556938 | 2.685147 | 1.713293 | 4.057498 | 280.75399 | 206.122576 | 371.809342 |
| 2002 | 283.614371 | 208.134526 | 375.317214 | 927.041027 | 728.046297 | 1193.273233 | 2.276194 | 1.469245 | 3.418946 | 258.623376 | 190.087201 | 341.424052 |
| 2003 | 280.241742 | 205.791066 | 370.702134 | 896.680236 | 702.745966 | 1155.85135 | 1.730233 | 1.120574 | 2.608295 | 229.887794 | 167.178588 | 303.119321 |
| 2004 | 276.624047 | 202.97777 | 365.806696 | 864.915955 | 675.840614 | 1117.188342 | 1.404074 | 0.919062 | 2.105967 | 210.015218 | 152.474179 | 277.474584 |
| 2005 | 273.071922 | 200.05107 | 361.351974 | 834.949223 | 650.146694 | 1081.362348 | 1.240619 | 0.815579 | 1.828612 | 196.929242 | 141.795199 | 259.641363 |
| 2006 | 268.04017 | 196.043958 | 354.581618 | 799.693027 | 620.544885 | 1037.502585 | 1.124656 | 0.737317 | 1.658832 | 184.00415 | 131.006428 | 244.178841 |
| 2007 | 260.846445 | 190.288857 | 345.539729 | 755.808081 | 582.856799 | 984.098304 | 1.046935 | 0.689403 | 1.533833 | 170.011487 | 120.806352 | 223.859636 |
| 2008 | 253.083614 | 183.953024 | 336.114433 | 711.973757 | 545.520049 | 929.373242 | 0.975179 | 0.646544 | 1.420795 | 156.066117 | 110.700709 | 205.754402 |
| 2009 | 246.338459 | 178.420504 | 328.455192 | 676.827155 | 516.064712 | 885.957321 | 0.905779 | 0.60077 | 1.321654 | 144.152346 | 102.005189 | 191.030337 |
| 2010 | 242.210059 | 175.191406 | 324.258843 | 659.002823 | 500.52007 | 865.108009 | 0.836268 | 0.556086 | 1.210089 | 136.4409 | 96.567462 | 181.135988 |
| 2011 | 239.883919 | 173.3285 | 321.386469 | 653.351864 | 496.058966 | 857.06048 | 0.779996 | 0.523234 | 1.1338 | 132.372508 | 93.673744 | 175.733767 |
| 2012 | 237.576943 | 171.388856 | 318.208022 | 648.974466 | 492.725554 | 850.968253 | 0.729875 | 0.493856 | 1.04954 | 128.922391 | 91.418629 | 171.425479 |
| 2013 | 235.452976 | 169.660781 | 315.492512 | 645.854709 | 490.332732 | 846.639833 | 0.673206 | 0.465894 | 0.955979 | 125.508218 | 88.282311 | 167.830014 |
| 2014 | 233.673778 | 168.163523 | 313.227423 | 643.961118 | 488.7806 | 844.246879 | 0.630534 | 0.439212 | 0.897538 | 122.899587 | 85.959382 | 163.500724 |
| 2015 | 232.402125 | 167.080537 | 311.777705 | 643.255144 | 488.349059 | 843.610413 | 0.600114 | 0.418347 | 0.848534 | 121.247701 | 84.949907 | 162.039328 |
| 2016 | 232.205324 | 167.01087 | 311.728961 | 648.53375 | 493.517333 | 849.377798 | 0.582978 | 0.404971 | 0.816865 | 120.65032 | 84.571337 | 160.903386 |
| 2017 | 233.159612 | 167.556708 | 313.210554 | 661.57347 | 504.408692 | 866.165316 | 0.574029 | 0.395756 | 0.819174 | 121.238923 | 84.794026 | 161.322411 |
| 2018 | 234.682392 | 168.522124 | 315.546324 | 677.769568 | 515.981941 | 887.549768 | 0.570154 | 0.391254 | 0.826295 | 122.725932 | 85.949406 | 164.008132 |
| 2019 | 236.185841 | 169.581491 | 318.101353 | 692.482494 | 527.921951 | 906.093585 | 0.558436 | 0.381061 | 0.809022 | 124.07518 | 86.933699 | 166.443646 |
| 2020 | 237.080878 | 170.222955 | 319.439038 | 701.050461 | 535.539599 | 916.902711 | 0.557411 | 0.37508 | 0.816241 | 125.501277 | 87.895697 | 168.22756 |
| 2021 | 239.199848 | 171.781598 | 322.447763 | 711.883001 | 541.947433 | 929.488744 | 0.599253 | 0.382987 | 0.900905 | 129.931974 | 90.56095 | 174.157558 |
| 2022 | 241.265804 | 172.789858 | 326.322765 | 720.884508 | 547.405383 | 942.522767 | 0.670968 | 0.393019 | 1.073258 | 135.106573 | 94.059411 | 181.226326 |
| 2023 | 240.642481 | 171.97616 | 324.384787 | 717.312867 | 544.87873 | 937.692549 | 0.661417 | 0.367356 | 1.090694 | 133.997049 | 93.136966 | 180.353528 |
| 2024 | 244.472691 | 174.354389 | 330.144695 | 749.49119 | 567.068021 | 981.715349 | 0.613644 | 0.359232 | 0.991173 | 135.634833 | 94.019307 | 183.255985 |
| 2025 | 246.375805 | 175.431894 | 332.837302 | 763.568703 | 576.639521 | 1000.692675 | 0.616399 | 0.353188 | 1.010956 | 137.732815 | 95.296887 | 186.466718 |
| 2026 | 248.275091 | 176.507083 | 335.524142 | 777.644022 | 586.209496 | 1019.662325 | 0.619196 | 0.347162 | 1.030803 | 139.834394 | 96.577987 | 189.683145 |
| 2027 | 250.455819 | 177.781837 | 338.498775 | 791.849247 | 595.858337 | 1038.158883 | 0.621858 | 0.343405 | 1.046773 | 142.353776 | 98.140991 | 193.375509 |
| 2028 | 252.633113 | 179.05423 | 341.469095 | 806.023893 | 605.48384 | 1056.61213 | 0.624537 | 0.339653 | 1.062773 | 144.870841 | 99.702901 | 197.064725 |
| 2029 | 254.805979 | 180.324027 | 344.433591 | 820.181118 | 615.097059 | 1075.038293 | 0.627292 | 0.33594 | 1.078885 | 147.388686 | 101.266558 | 200.754405 |
| 2030 | 256.976935 | 181.593024 | 347.395257 | 834.32144 | 624.696956 | 1093.441039 | 0.630103 | 0.332254 | 1.095087 | 149.906518 | 102.831267 | 204.443604 |
| 2031 | 259.147942 | 182.862519 | 350.356488 | 848.443076 | 634.280823 | 1111.820929 | 0.63294 | 0.328577 | 1.111335 | 152.422774 | 104.39547 | 208.130586 |
| 2032 | 261.126451 | 184.013552 | 353.144863 | 859.163062 | 641.665544 | 1125.476055 | 0.634641 | 0.326235 | 1.121844 | 154.57625 | 105.76056 | 211.257177 |
| 2033 | 263.107211 | 185.166268 | 355.935053 | 869.848536 | 649.020702 | 1139.090886 | 0.636312 | 0.323871 | 1.13232 | 156.724597 | 107.121545 | 214.377125 |
| 2034 | 265.088039 | 186.319475 | 358.724762 | 880.495946 | 656.34516 | 1152.660217 | 0.638002 | 0.321511 | 1.142836 | 158.868539 | 108.479601 | 217.490522 |
| 2035 | 267.06956 | 187.473257 | 361.51504 | 891.120872 | 663.650719 | 1166.203512 | 0.63972 | 0.319162 | 1.153404 | 161.009824 | 109.835885 | 220.600035 |
| 2036 | 269.051988 | 188.627322 | 364.306468 | 901.737984 | 670.948566 | 1179.738774 | 0.641453 | 0.316818 | 1.164003 | 163.149697 | 111.190999 | 223.707823 |
| 2037 | 270.875264 | 189.673209 | 366.894029 | 908.130167 | 675.25388 | 1188.023126 | 0.641597 | 0.315463 | 1.167637 | 164.491063 | 112.051458 | 225.651934 |
| 2038 | 272.700682 | 190.719149 | 369.484305 | 914.524048 | 679.558763 | 1196.310305 | 0.641712 | 0.314091 | 1.171229 | 165.83133 | 112.910217 | 227.595089 |
| 2039 | 274.527438 | 191.765151 | 372.076474 | 920.904095 | 683.851586 | 1204.582597 | 0.641829 | 0.312718 | 1.174832 | 167.168749 | 113.7664 | 229.534235 |
| 2040 | 276.354395 | 192.81044 | 374.669345 | 927.278959 | 688.139252 | 1212.849904 | 0.641956 | 0.311348 | 1.178456 | 168.50458 | 114.62113 | 231.471407 |
| **India** | | | | | | | | | | | | |
| 1994 | 147.276063 | 98.868573 | 211.29912 | 474.104375 | 327.27695 | 681.583396 | 0.665363 | 0.380852 | 1.095609 | 85.221486 | 59.909616 | 115.650647 |
| 1995 | 147.229815 | 98.8291 | 211.481702 | 474.316103 | 326.395316 | 683.047143 | 0.674123 | 0.387012 | 1.091182 | 85.732002 | 60.242136 | 115.601636 |
| 1996 | 147.214085 | 99.189685 | 210.863649 | 474.933617 | 327.392855 | 681.38151 | 0.703494 | 0.410337 | 1.137201 | 87.515319 | 61.370141 | 117.593086 |
| 1997 | 147.290537 | 99.481766 | 210.153201 | 476.459429 | 330.006617 | 679.548403 | 0.737953 | 0.428466 | 1.184413 | 90.256815 | 63.082964 | 121.724822 |
| 1998 | 147.402153 | 100.042958 | 209.018839 | 478.270641 | 333.360519 | 677.949042 | 0.764237 | 0.436926 | 1.244603 | 93.169353 | 65.252763 | 124.416877 |
| 1999 | 147.492446 | 100.311667 | 208.457596 | 479.743141 | 335.586125 | 677.458613 | 0.779267 | 0.444741 | 1.275156 | 95.35482 | 66.245491 | 127.782764 |
| 2000 | 147.504732 | 100.318701 | 208.294582 | 480.259158 | 334.163428 | 677.28763 | 0.790857 | 0.451381 | 1.310905 | 96.547135 | 66.969993 | 129.464324 |
| 2001 | 147.254786 | 100.097923 | 207.980041 | 478.846719 | 332.949396 | 675.623387 | 0.804188 | 0.464997 | 1.320971 | 96.487288 | 67.121585 | 130.09241 |
| 2002 | 146.685019 | 99.593565 | 207.328843 | 475.479212 | 329.964378 | 671.940806 | 0.818606 | 0.477029 | 1.322749 | 95.49948 | 66.415811 | 128.627422 |
| 2003 | 145.930913 | 98.92501 | 206.470302 | 470.995271 | 325.977536 | 667.16525 | 0.794724 | 0.470491 | 1.262962 | 92.703458 | 64.731263 | 124.781344 |
| 2004 | 145.127874 | 98.19631 | 205.570643 | 466.22779 | 321.767156 | 662.143181 | 0.772869 | 0.463887 | 1.225986 | 89.902288 | 63.081908 | 120.994031 |
| 2005 | 144.41122 | 97.577276 | 204.757748 | 462.003814 | 318.084819 | 657.829418 | 0.818508 | 0.498711 | 1.297881 | 89.9119 | 63.82218 | 120.14814 |
| 2006 | 143.835939 | 97.125481 | 204.240021 | 459.193266 | 316.266772 | 655.729269 | 0.734838 | 0.454649 | 1.151602 | 84.818423 | 59.991368 | 113.298804 |
| 2007 | 143.38343 | 96.593467 | 203.922952 | 457.787316 | 313.987457 | 657.067805 | 0.709753 | 0.437288 | 1.110829 | 81.722425 | 57.703628 | 110.038793 |
| 2008 | 143.079824 | 96.098619 | 204.066649 | 457.320628 | 312.156806 | 657.944934 | 0.702114 | 0.435735 | 1.096938 | 79.34722 | 57.394758 | 105.944451 |
| 2009 | 142.951734 | 95.724865 | 204.647664 | 457.325305 | 310.694036 | 662.119088 | 0.64191 | 0.394324 | 1.002354 | 75.679527 | 54.433178 | 102.037648 |
| 2010 | 143.025583 | 95.622322 | 205.207338 | 457.332054 | 309.593326 | 664.188235 | 0.605731 | 0.376873 | 0.941171 | 73.660199 | 53.093953 | 98.488322 |
| 2011 | 143.88837 | 96.364316 | 206.180071 | 459.824669 | 312.040847 | 666.394769 | 0.573554 | 0.359325 | 0.880033 | 73.576909 | 53.003601 | 97.708679 |
| 2012 | 145.734876 | 97.917097 | 208.337945 | 466.068567 | 318.058462 | 672.302507 | 0.562183 | 0.35383 | 0.853209 | 75.689375 | 54.492751 | 100.824576 |
| 2013 | 148.002988 | 99.755959 | 210.981835 | 473.772871 | 325.337175 | 679.684342 | 0.522749 | 0.335488 | 0.777693 | 77.446817 | 55.819375 | 102.734186 |
| 2014 | 150.132789 | 101.474753 | 213.545191 | 480.647964 | 331.797855 | 686.346159 | 0.502378 | 0.325452 | 0.743007 | 79.569243 | 57.140007 | 106.004774 |
| 2015 | 151.567696 | 102.575947 | 215.201867 | 484.404644 | 335.311903 | 690.17691 | 0.491643 | 0.319944 | 0.72287 | 80.634084 | 57.747095 | 107.077448 |
| 2016 | 152.353586 | 103.763235 | 215.390278 | 484.37092 | 337.864486 | 688.667318 | 0.499568 | 0.32476 | 0.736825 | 81.705069 | 58.483759 | 108.884895 |
| 2017 | 152.983335 | 104.739328 | 215.064846 | 482.344028 | 339.63004 | 683.678178 | 0.49397 | 0.316329 | 0.734565 | 82.590062 | 59.17545 | 109.402203 |
| 2018 | 153.570394 | 105.81456 | 214.33427 | 479.730132 | 340.668983 | 678.836584 | 0.495484 | 0.315706 | 0.74626 | 84.190569 | 60.388776 | 111.47661 |
| 2019 | 154.224635 | 106.682753 | 214.771302 | 477.931758 | 341.417217 | 674.411476 | 0.514053 | 0.327799 | 0.780206 | 86.398471 | 62.341528 | 114.285503 |
| 2020 | 155.055632 | 107.10136 | 216.019489 | 478.354014 | 341.717785 | 674.822982 | 0.537232 | 0.339747 | 0.820884 | 88.087544 | 62.744509 | 116.896382 |
| 2021 | 156.820053 | 108.431871 | 218.048355 | 483.846218 | 346.37773 | 680.779275 | 0.602568 | 0.367701 | 0.940111 | 92.915442 | 66.257033 | 123.035583 |
| 2022 | 158.220657 | 109.530719 | 219.780471 | 488.273535 | 350.205952 | 685.537979 | 0.595514 | 0.340152 | 0.968448 | 94.262112 | 66.664523 | 125.492886 |
| 2023 | 156.380926 | 108.184884 | 217.318475 | 476.234045 | 341.467664 | 665.587343 | 0.617646 | 0.33872 | 1.040583 | 94.394209 | 66.650023 | 126.513866 |
| 2024 | 156.747722 | 109.63093 | 217.092132 | 475.24961 | 345.986006 | 666.424228 | 0.631838 | 0.360858 | 1.046031 | 96.772837 | 68.805881 | 128.774524 |
| 2025 | 156.918003 | 110.158018 | 217.007698 | 473.211042 | 346.466447 | 662.962806 | 0.650719 | 0.366735 | 1.09001 | 98.587474 | 70.072184 | 131.226105 |
| 2026 | 157.088451 | 110.685249 | 216.922141 | 471.161621 | 346.938239 | 659.487177 | 0.669597 | 0.37261 | 1.13399 | 100.400309 | 71.337129 | 133.675009 |
| 2027 | 157.072828 | 111.040406 | 216.673591 | 468.947364 | 346.782829 | 656.488884 | 0.685183 | 0.376982 | 1.171965 | 101.635119 | 72.255211 | 135.444571 |
| 2028 | 157.055287 | 111.394761 | 216.421591 | 466.723656 | 346.62 | 653.481123 | 0.700788 | 0.381363 | 1.209981 | 102.867976 | 73.171773 | 137.211476 |
| 2029 | 157.037294 | 111.748832 | 216.167576 | 464.492774 | 346.450221 | 650.465613 | 0.716418 | 0.385754 | 1.248047 | 104.099182 | 74.086852 | 138.976049 |
| 2030 | 157.019393 | 112.102818 | 215.91254 | 462.256136 | 346.274776 | 647.44301 | 0.732057 | 0.390149 | 1.286133 | 105.329274 | 75.0009 | 140.73893 |
| 2031 | 157.001274 | 112.456568 | 215.656511 | 460.014213 | 346.094942 | 644.41363 | 0.74769 | 0.394543 | 1.324216 | 106.558476 | 75.914271 | 142.500454 |
| 2032 | 156.775745 | 112.591851 | 215.250712 | 458.119755 | 345.74218 | 641.924525 | 0.758754 | 0.397375 | 1.351858 | 107.354907 | 76.53535 | 143.69529 |
| 2033 | 156.548018 | 112.725818 | 214.842206 | 456.218584 | 345.386202 | 639.426911 | 0.769817 | 0.400206 | 1.379503 | 108.150433 | 77.156135 | 144.888853 |
| 2034 | 156.319837 | 112.859264 | 214.432479 | 454.313609 | 345.027098 | 636.924293 | 0.780882 | 0.403036 | 1.407156 | 108.94508 | 77.776334 | 146.081057 |
| 2035 | 156.091863 | 112.992549 | 214.022345 | 452.405223 | 344.664848 | 634.416207 | 0.791941 | 0.405865 | 1.434799 | 109.739434 | 78.396322 | 147.272549 |
| 2036 | 155.863983 | 113.12568 | 213.611829 | 450.492979 | 344.29956 | 631.901667 | 0.802997 | 0.408695 | 1.462437 | 110.533869 | 79.016434 | 148.463854 |
| 2037 | 155.26088 | 112.957631 | 212.888938 | 448.758128 | 343.716731 | 629.640434 | 0.808201 | 0.40966 | 1.475638 | 110.897585 | 79.319037 | 149.04982 |
| 2038 | 154.656273 | 112.788913 | 212.164333 | 447.018775 | 343.132039 | 627.372806 | 0.813432 | 0.410641 | 1.488888 | 111.261838 | 79.622331 | 149.636594 |
| 2039 | 154.051941 | 112.620405 | 211.439649 | 445.278227 | 342.546176 | 625.103509 | 0.818685 | 0.411635 | 1.502182 | 111.62648 | 79.925946 | 150.223922 |
| 2040 | 153.448439 | 112.452244 | 210.715441 | 443.536477 | 341.958603 | 622.83164 | 0.823943 | 0.412632 | 1.515483 | 111.991334 | 80.229592 | 150.811353 |
| **the United States** | | | | | | | | | | | | |
| 1994 | 494.115789 | 348.189184 | 692.466091 | 2390.226278 | 1865.103996 | 3056.334334 | 3.840915 | 2.822789 | 5.248504 | 482.6147 | 363.478954 | 601.152646 |
| 1995 | 495.436787 | 349.596169 | 694.03238 | 2389.558429 | 1869.276256 | 3053.57491 | 4.197459 | 3.096609 | 5.648703 | 501.891732 | 377.860511 | 624.108551 |
| 1996 | 496.187966 | 351.498078 | 694.730445 | 2387.802729 | 1876.199609 | 3044.743108 | 4.346341 | 3.224921 | 5.907686 | 512.012648 | 387.992663 | 635.205913 |
| 1997 | 496.357258 | 353.041797 | 693.740462 | 2383.727658 | 1880.774 | 3035.012437 | 4.517993 | 3.361334 | 6.079759 | 525.097289 | 399.686096 | 651.085196 |
| 1998 | 496.604406 | 353.683885 | 692.016868 | 2382.285411 | 1887.135437 | 3027.654572 | 4.936559 | 3.665598 | 6.581581 | 550.6636 | 420.452495 | 682.005649 |
| 1999 | 497.591337 | 354.648412 | 692.976081 | 2388.429946 | 1898.528218 | 3029.517524 | 5.577903 | 4.153411 | 7.413951 | 587.870421 | 453.281206 | 725.577964 |
| 2000 | 499.975049 | 356.362031 | 695.967769 | 2407.014765 | 1920.180525 | 3047.344708 | 6.253503 | 4.651083 | 8.2311 | 628.657182 | 487.921087 | 777.407173 |
| 2001 | 503.609134 | 359.700532 | 699.399199 | 2437.296418 | 1952.304957 | 3078.582069 | 7.012935 | 5.202449 | 9.232169 | 674.643426 | 527.973006 | 835.953116 |
| 2002 | 507.670481 | 363.1345 | 703.719202 | 2472.179382 | 1988.625801 | 3114.930192 | 7.97985 | 5.903287 | 10.453722 | 731.161881 | 576.239602 | 902.534006 |
| 2003 | 512.027715 | 366.948628 | 708.410157 | 2510.34942 | 2026.915037 | 3155.294215 | 8.892743 | 6.542325 | 11.579956 | 786.209694 | 617.632326 | 972.636618 |
| 2004 | 516.584639 | 370.849224 | 713.530684 | 2550.864905 | 2067.634943 | 3196.082948 | 9.618877 | 7.02408 | 12.536914 | 833.687229 | 651.478697 | 1033.026993 |
| 2005 | 521.263752 | 374.992471 | 719.056258 | 2592.596047 | 2109.549451 | 3238.549459 | 10.561848 | 7.69463 | 13.780714 | 891.41953 | 694.894528 | 1105.270919 |
| 2006 | 527.243425 | 380.153552 | 725.652302 | 2641.629861 | 2152.925093 | 3293.524574 | 11.449979 | 8.243681 | 14.995518 | 949.119817 | 737.815548 | 1173.026201 |
| 2007 | 535.35681 | 387.699262 | 735.275832 | 2703.781661 | 2205.849435 | 3367.298643 | 12.015768 | 8.724321 | 15.695874 | 994.718805 | 770.638108 | 1227.876454 |
| 2008 | 544.524782 | 396.424875 | 746.382208 | 2774.691247 | 2264.736321 | 3453.161961 | 12.557124 | 9.307266 | 16.292435 | 1041.31053 | 802.365137 | 1281.787866 |
| 2009 | 553.733713 | 404.579405 | 757.862229 | 2849.985798 | 2328.10416 | 3543.59271 | 13.038281 | 9.60427 | 16.879284 | 1087.639886 | 848.086317 | 1331.599844 |
| 2010 | 561.988758 | 411.71469 | 767.432573 | 2925.64421 | 2393.351562 | 3629.01342 | 13.597458 | 10.005677 | 17.617229 | 1140.433872 | 897.019766 | 1390.653909 |
| 2011 | 571.522138 | 420.153979 | 777.858905 | 3017.129967 | 2475.702434 | 3725.870886 | 14.355242 | 10.591759 | 18.588065 | 1211.16293 | 962.296241 | 1473.760738 |
| 2012 | 584.16997 | 431.50684 | 792.130002 | 3136.38301 | 2582.185474 | 3854.009272 | 15.020027 | 11.242779 | 19.517181 | 1289.724495 | 1027.363467 | 1563.976696 |
| 2013 | 598.511278 | 443.853142 | 808.610352 | 3275.86882 | 2706.294255 | 4008.175456 | 15.967497 | 12.113559 | 20.736447 | 1389.176304 | 1109.485576 | 1685.799492 |
| 2014 | 613.028992 | 455.839683 | 825.362237 | 3427.133243 | 2836.947012 | 4183.102515 | 17.224489 | 13.193513 | 22.096061 | 1508.764038 | 1207.735951 | 1822.730924 |
| 2015 | 626.303731 | 467.111162 | 841.099096 | 3582.627817 | 2973.036948 | 4363.562397 | 19.120028 | 14.730107 | 24.491304 | 1660.256307 | 1335.99694 | 2015.200494 |
| 2016 | 640.978096 | 480.417133 | 856.695693 | 3786.895314 | 3163.763137 | 4579.162941 | 21.568938 | 16.582418 | 27.519785 | 1852.389971 | 1497.561205 | 2240.879924 |
| 2017 | 658.973033 | 496.628097 | 875.971002 | 4056.762987 | 3414.303608 | 4867.175144 | 23.535366 | 18.097761 | 30.06188 | 2034.323828 | 1645.053985 | 2455.395895 |
| 2018 | 677.820594 | 514.068755 | 896.756544 | 4343.962513 | 3680.486946 | 5171.1853 | 24.53323 | 18.840651 | 31.192347 | 2170.18178 | 1754.164291 | 2618.984559 |
| 2019 | 695.006101 | 529.435128 | 914.577971 | 4600.34218 | 3914.904489 | 5449.60028 | 26.567985 | 20.195887 | 33.8964 | 2342.780563 | 1898.504633 | 2827.994711 |
| 2020 | 708.137445 | 540.21559 | 927.53999 | 4779.216158 | 4075.905299 | 5645.136657 | 31.25503 | 23.730422 | 40.307622 | 2611.323103 | 2106.71438 | 3163.597969 |
| 2021 | 721.773243 | 551.409124 | 942.798483 | 4914.321585 | 4192.077082 | 5799.011912 | 35.055727 | 26.386494 | 45.520815 | 2814.346176 | 2258.330406 | 3420.582962 |
| 2022 | 730.624044 | 558.358113 | 956.089317 | 4992.249121 | 4257.319129 | 5892.006377 | 36.902735 | 27.889405 | 47.590059 | 2907.110887 | 2336.049747 | 3524.822705 |
| 2023 | 730.388565 | 555.684172 | 960.096853 | 5001.727978 | 4257.402318 | 5915.053349 | 36.41835 | 27.775614 | 46.728508 | 2879.855965 | 2321.324767 | 3482.453196 |
| 2024 | 747.391012 | 571.313646 | 976.442585 | 5366.036387 | 4605.259376 | 6292.07774 | 42.626433 | 32.014426 | 55.203241 | 3500.167058 | 2657.801938 | 4005.550431 |
| 2025 | 757.378701 | 579.346687 | 988.402296 | 5535.486469 | 4760.504593 | 6476.006859 | 45.786555 | 34.310139 | 59.376735 | 3698.305159 | 2816.496692 | 4247.476825 |
| 2026 | 767.366301 | 587.379641 | 1000.359874 | 5704.929772 | 4915.744711 | 6659.921115 | 48.948751 | 36.607326 | 63.552933 | 3868.317066 | 2975.252551 | 4489.490294 |
| 2027 | 777.58069 | 595.491149 | 1012.543203 | 5845.127652 | 5041.050325 | 6814.554438 | 51.999802 | 38.800462 | 67.605111 | 4038.369743 | 3111.555257 | 4697.033253 |
| 2028 | 787.7938 | 603.601833 | 1024.723357 | 5985.301485 | 5166.332782 | 6969.157577 | 55.052439 | 40.994655 | 71.659415 | 4208.47648 | 3247.891343 | 4904.622958 |
| 2029 | 798.004102 | 611.71011 | 1036.898768 | 6125.43703 | 5291.580126 | 7123.71565 | 58.107145 | 43.190286 | 75.716421 | 4378.639402 | 3384.272614 | 5112.273938 |
| 2030 | 808.212286 | 619.816302 | 1049.071131 | 6265.537 | 5416.796465 | 7278.230095 | 61.163947 | 45.387365 | 79.776181 | 4548.854406 | 3520.700921 | 5319.988251 |
| 2031 | 818.419334 | 627.921137 | 1061.242439 | 6405.604719 | 5541.986636 | 7432.702247 | 64.222702 | 47.585768 | 83.838538 | 4667.722638 | 3657.172902 | 5527.762495 |
| 2032 | 826.151128 | 634.065567 | 1070.358837 | 6500.56728 | 5625.860764 | 7537.681128 | 66.612247 | 49.293312 | 87.021355 | 4786.608045 | 3752.412412 | 5672.859172 |
| 2033 | 833.88164 | 640.209187 | 1079.474485 | 6595.491223 | 5709.704244 | 7642.610048 | 69.002932 | 51.001562 | 90.205788 | 4905.517645 | 3847.666746 | 5817.975219 |
| 2034 | 841.609409 | 646.350612 | 1088.587213 | 6690.356994 | 5793.497956 | 7747.469976 | 71.395249 | 52.710915 | 93.392404 | 5024.4558 | 3942.942691 | 5963.118257 |
| 2035 | 849.335224 | 652.490269 | 1097.697676 | 6785.17322 | 5877.249078 | 7852.271102 | 73.789193 | 54.42137 | 96.581199 | 5143.421371 | 4038.24326 | 6108.292376 |
| 2036 | 857.060584 | 658.629173 | 1106.807589 | 6879.951562 | 5960.967834 | 7957.0268 | 76.184584 | 56.132783 | 99.771955 | 5198.853221 | 4133.566694 | 6253.497127 |
| 2037 | 861.13458 | 661.82953 | 1111.679507 | 6923.808023 | 5999.636357 | 8004.806833 | 77.466256 | 57.050313 | 101.474231 | 5254.27877 | 4178.120869 | 6320.289536 |
| 2038 | 865.209019 | 665.029981 | 1116.552601 | 6967.63323 | 6038.280419 | 8052.54783 | 78.748242 | 57.967977 | 103.177046 | 5309.701743 | 4222.668683 | 6387.073316 |
| 2039 | 869.283762 | 668.230456 | 1121.427131 | 7011.402675 | 6076.877064 | 8100.225678 | 80.03109 | 58.886197 | 104.881064 | 3302.105031 | 4267.214872 | 6453.8532 |
| 2040 | 873.358496 | 671.430737 | 1126.302453 | 7055.123769 | 6115.432271 | 8147.848296 | 81.314703 | 59.804903 | 106.586153 | 5365.123872 | 4311.761164 | 6520.62962 |

ASIR: age-standardized incidence rate, ASPR: age-standardized prevalence rate, ASMR: age-standardized mortality rate, ASDR: age-standardized DALYs rate. DALYs: disability-adjusted life years.

Supplementary Figure S1. The average annual percent change (AAPC) of incidence, prevalence, deaths, and disability-adjusted life years (DALYs) for drug use disorders, from 1990 to 2023, in China (A), India (B), and the United States (C), stratified by age and sex.


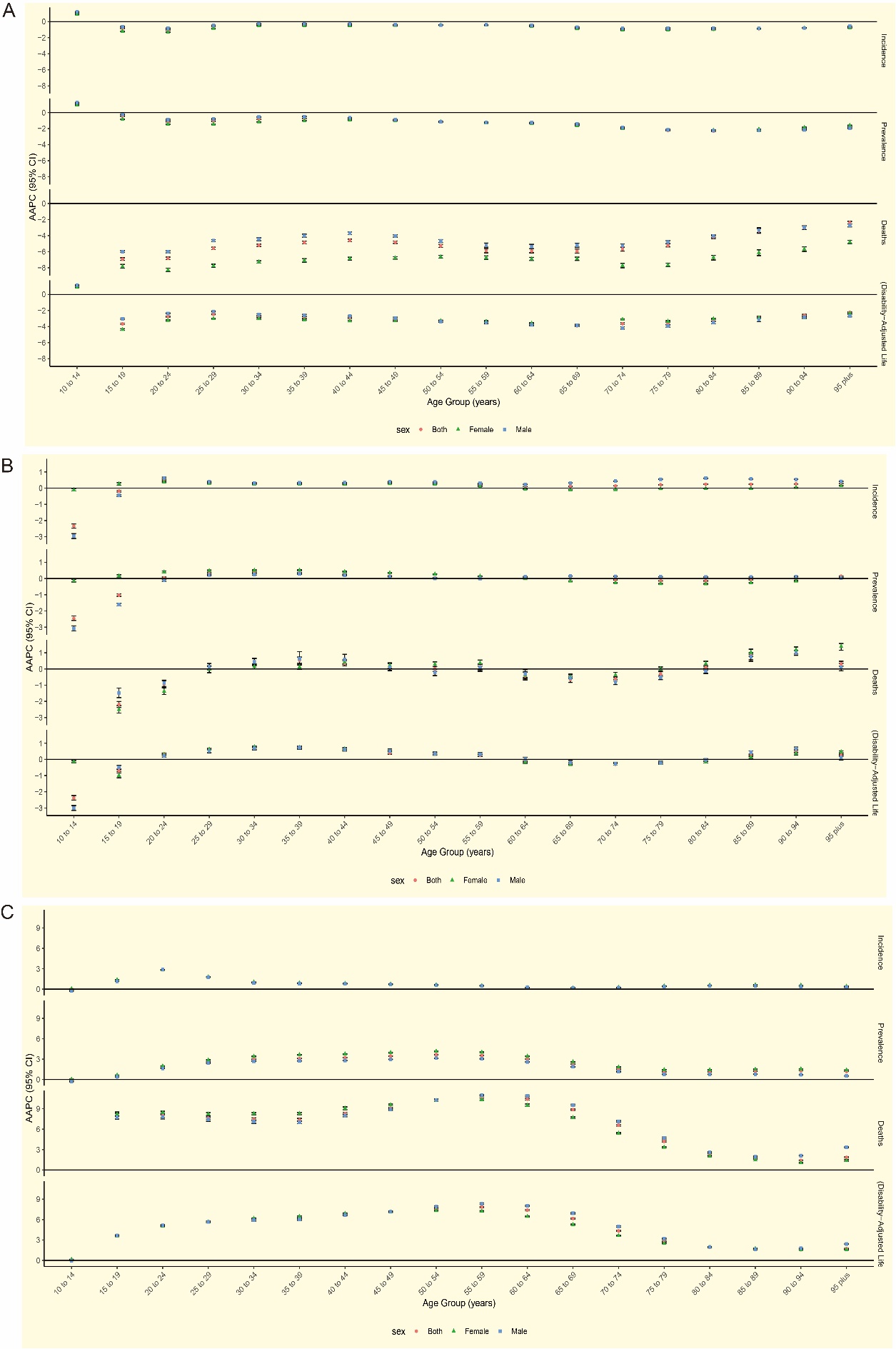

Supplement: Supplementary file 1 [file DataSheet1.docx]
